# Gaultherin, a Natural Alternative to Aspirin: A Comprehensive Review of Molecular Mechanisms, Pharmacokinetics, Biocompatibility, Isolation Techniques, and Plant Sources

**DOI:** 10.3390/ijms26157280

**Published:** 2025-07-28

**Authors:** Piotr Michel

**Affiliations:** Department of Pharmacognosy, Faculty of Pharmacy, Medical University of Lodz, Muszyńskiego 1, 90-151 Lodz, Poland; piotr.michel@umed.lodz.pl; Tel.: +48-42-677-9169

**Keywords:** gaultherin, monotropitoside, monotropitin, molecular mechanisms, toxicology, pharmacokinetics, plant materials

## Abstract

Gaultherin [methyl salicylate 2-*O*-β-D-xylopyranosyl-(1→6)-β-D-glucopyranoside] is a natural salicylate found in some plant species belonging primarily to the Ericaceae and Rosaceae families. Biological studies conducted since the beginning of the 21st century have suggested the potential use of gaultherin in treating various diseases related to inflammation and oxidative stress, including rheumatoid arthritis, sciatica, neuralgia, and muscular pain. The accumulated results indicated a targeted range of biological effects, particularly anti-inflammatory, antipyretic, and anti-rheumatic properties associated with reduced adverse outcomes. The molecular mechanisms involve the influence on several signalling pathways, including NF-κB, MAPK, and potentially AMPK, as well as the inhibition of critical pro-inflammatory enzymes, such as COX-2. This inhibition is achieved without affecting the COX-1 isoform, thereby preventing side effects such as bleeding ulcers or intracranial haemorrhage. This overview summarises the current knowledge about pharmacokinetics, molecular mechanisms, pharmacology, and biocompatibility of gaultherin. Additionally, four methods for isolating gaultherin from plant material and its distribution within the plant kingdom were the focal points of review and discussion. The paper also describes significant differences between synthetic aspirin and natural gaultherin in their biological potential and side effects, resulting from their different mechanisms of action. As a prodrug of salicylic acid, gaultherin releases salicylic acid gradually through enzymatic hydrolysis in the gastrointestinal tract. This controlled release minimises direct gastric irritation and accounts for its superior gastrointestinal safety profile compared to aspirin. Unlike aspirin, which irreversibly inhibits COX-1 and can lead to serious side effects with chronic use, gaultherin selectively inhibits COX-2 while sparing COX-1. These properties position gaultherin as a compelling natural alternative for patients requiring long-term anti-inflammatory therapy with reduced risk of gastrointestinal or bleeding complications.

## 1. Introduction

The history of natural salicylates dates back to approximately 1550 BC, when dried myrtle leaf powder was recorded as an analgesic agent in the Ebers Papyrus, the oldest known medical text from ancient Egypt. This text is likely a copy of a manuscript influenced by Sumerian medical practice from around 3000 BC [1]. The Assyrians also used willow leaf extract to alleviate vascular and muscle pain [2]. In the 5th century BC, the renowned Ancient Greek physician Hippocrates recommended that patients chew willow bark to relieve fever and pain [3]. Similarly, one of the four classics of Traditional Chinese Medicine, Huang Di Nei Jing, recorded willow bark as an external application for toothaches [4]. However, in ancient times, people used these herbs to treat fever and pain without realising that salicylates were the key active compounds responsible for their therapeutic effects. In 1763, Reverend Edward Stone was the first to identify that willow bark extract contained salicylic acid, which helped relieve pain and shivering. The active prodrug of salicylic acid was recognised as an ingredient in willow bark in 1828 when Johann Buchner refined it into yellow crystals and named it salicin, after the willow tree’s genus, *Salix* [4]. Further studies revealed that myrtle leaves, willow, and poplar bark contained further methyl salicylate glycosides [5]. With advancements in phytochemical studies, the researchers have established that salicylates are widely distributed across various plant species [6].

In recent years, scientists have paid increasing attention to plant-derived compounds for their potential in biomedical applications, particularly as safer, multi-targeted alternatives to conventional synthetic drugs. Numerous studies have highlighted the therapeutic relevance of natural phytochemicals, including polyphenols, alkaloids, and terpenoids, for their anti-inflammatory, antioxidant, and anticancer properties. Among these, prunin (naringenin glucoside), a naturally occurring flavonoid glycoside, has emerged as a promising anticancer agent with additional antioxidant and immunomodulatory benefits, mediated through key molecular pathways such as the PI3K/Akt/mTOR, NF-κB, and MAPKs [7]. Similarly, juglanin (kaempferol arabinoside), another plant flavonoid, has been shown to exert anti-inflammatory, antioxidative, and anticancer properties through modulation of AMPK, MAPK, and NF-κB pathways, with recent reviews underscoring its therapeutic versatility and potential in complex diseases [8]. Enzymatically modified isoquercitrin (quercetin glucoside) is also gaining attention for its superior bioavailability and broad-spectrum biological effects, including cardioprotective, neuroprotective, and chemopreventive actions, highlighting the benefits of molecular modification in enhancing the bioactivity of plant-derived compounds [9]. These studies collectively exemplify the renewed interest and expanding evidence base for plant-derived compounds in modern biomedical applications. Methyl salicylate glycosides, including gaultherin and its analogues, have likewise drawn renewed attention for their potent anti-inflammatory and analgesic properties, offering a promising natural alternative to synthetic nonsteroidal anti-inflammatory drugs.

**Figure 1:**
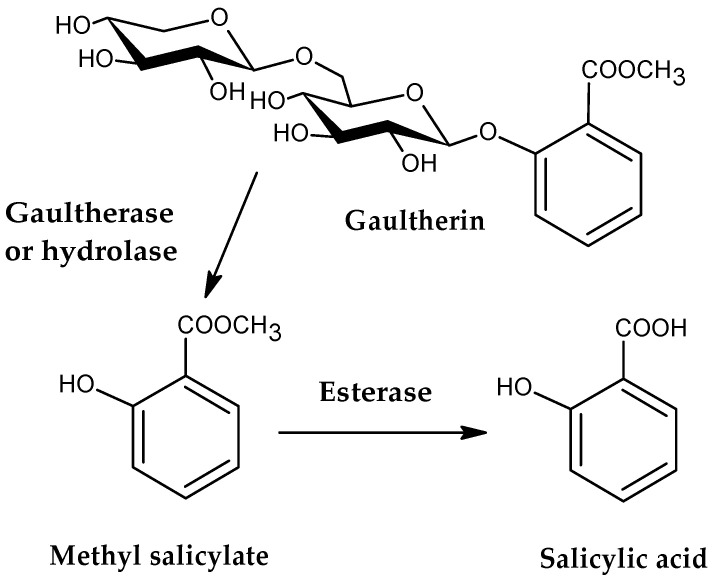
The chemical structure of gaultherin and its metabolic pathway.

Gaultherin [methyl salicylate 2-*O*-β-D-xylopyranosyl-(1→6)-β-D-glucopyranoside; CAS 490-67-5, Figure 1], formerly known as monotropitin or monotropitoside [10,11], is a member of an important group of naturally occurring methyl salicylate glycosides [6], and a compound that has gained significant attention in recent years as an active anti-inflammatory and analgesic agent [12,13]. This natural salicylate releases methyl salicylate on mild, enzymatically induced hydrolysis when the plant material is damaged [14]. Researchers have identified gaultherin in various plants, including *Betula lenta* L. and *Filipendula* Mill. species, as well as *Monotropa* L. and *Gaultheria* L. plants, from which subsequent chemical compound names were derived. Next to the two methyl salicylate triglycosides {MSTG-A, i.e., methyl salicylate 2-*O*-β-D-xylopyranosyl-(1→2)-[β-D-xylopyranosyl-(1→6)]-β-D-glucopyranoside, and MSTG-B, i.e., methyl salicylate 2-*O*-β-D-glucopyranosyl-(1→2)-[β-D-xylopyranosyl-(1→6)]-β-D-glucopyranoside} and methyl salicylate 2-*O*-β-D-lactoside (MSL), gaultherin is one of the most prevalent natural salicylates in the plant kingdom.

The study of gaultherin’s biological activity dates back to 2006 [15], but research has intensified significantly between 2019 and 2024. Most studies on in vivo animal models and in vitro cellular experiments have investigated its potential for treating various chronic diseases related to oxidative stress and inflammation. Gaultherin’s primary mechanisms of action comprise anti-inflammatory, analgesic, antipyretic, and antioxidant properties. The compound’s targeted signalling pathways, including its inhibitory effects on cyclooxygenase-2 and involvement in nuclear factor kappa-light-chain-enhancer of activated B cells (NF-κB), mitogen-activated protein kinases (MAPKs), and potentially AMP-activated protein kinase (AMPK), have been extensively studied, further supporting its potential therapeutic applications.

Over the past decade, a growing body of research has enhanced the understanding of the pharmacokinetics and therapeutic potential of gaultherin and related methyl salicylate glycosides. Recent studies have revealed the biotransformation pathways of methyl salicylate glycosides via gut microbiota from *Gaultheria* species, demonstrating the role of terminal sugar moieties in modulating their metabolic fate and pharmacokinetics [16]. In vitro and in situ models confirmed that MSTG-A, MSTG-B, and gaultherin exhibit favourable intestinal absorption characteristics and are subject to P-glycoprotein-mediated transport [17]. Building on these insights, a comprehensive PK-PD and metabolomics study identified spleen-targeted distribution and modulation of the immune-inflammatory pathway as central to their anti-rheumatic efficacy [18]. Complementing these mechanistic findings, gaultherin has been shown to offer aspirin-like anti-inflammatory effects without gastric toxicity, owing to its sustained gut-mediated release [15]. Earlier work also established the analgesic and peripheral anti-inflammatory effects of a salicylate-rich fraction containing MSTG-A, MSTG-B, and gaultherin [19]. More recently, the salicylate-rich fraction from *G. trichophylla* was shown to exhibit strong anti-inflammatory and analgesic activities through in vivo, in vitro, and in silico studies, including COX/LOX inhibition and molecular docking [20]. Furthermore, adjuvant-induced arthritis models demonstrated the therapeutic benefits of *G. trichophylla* in restoring hematological and biochemical parameters, reducing joint destruction, and targeting TNF-α via molecular docking and network pharmacology [21]. These studies reflect an ongoing interest in the therapeutic viability of gaultherin and other methyl salicylate glycosides, positioning them as promising candidates for safe, long-term anti-inflammatory therapy.

The present review aims to summarise over a century of biological research on gaultherin, focusing primarily on its significance as an anti-inflammatory, analgesic, and antipyretic agent. This work covers its distribution in the plant kingdom and isolation techniques from plant material. Furthermore, metabolism, cytocompatibility, and biological activity studies of gaultherin were reviewed, focusing on the main differences between the synthetic representative of nonsteroidal anti-inflammatory drugs (NSAIDs), i.e., aspirin, and the natural glycosidic analogues of methyl salicylate.

## 2. Occurrence in Plant Species

In 1844, Procter Jr. identified gaultherin as a conjugate of methyl salicylate and two monosaccharides, i.e., glucose and xylose. Still, scientists believed that gaultherin is absent from the plant it was named after, likely due to its instability [22]. Interest in gaultherin resurfaced nearly 80 years later. Gaultherin was first isolated in its pure form from *Monotropa hypopitys* L. and named by Bridel as monotropitin, after the plant genus [10]. The researchers subsequently changed the name from monotropitin to monotropitoside [11]. In 1928, Bridel and Grillon determined that monotropitoside was gaultherin and that gaultherin could only be extracted from *Gaultheria procumbens* L. [23]. The compound is a primveroside of methyl salicylate, which is hydrolysed by the specific plant enzyme gaultherase, yielding disaccharide primverose [β-D-xylopyranosyl-(1→6)-β-D-glucopyranose] [11,24,25,26,27]. In subsequent years, Bridel isolated gaultherin from other natural sources, including *Spiraea ulmaria* L., *S. filipendula* L., and *S. gigantea* var. *rosea* L. [28], *Betula lenta* L. [24,25], *Prunus amygdalus* Batsch [29], *Primula officinalis* L. [30], and *Gaultheria procumbens* L. [23,31]. A pair of British scientists performed the first chemical synthesis of gaultherin in 1931. Researchers have confirmed that during hydrolysis with 3% H_2_SO_4_, the compound forms 1 mol of methyl salicylate, 1 mol of D-glucose, and 1 mol of D-xylose [32]. The successful synthesis of methyl salicylate primveroside was also carried out in later years by two Russian researchers [33] and further by Chinese investigators [34].

Gaultherin was detected in over thirty plant species from nineteen botanical families, such as Apocynaceae Juss., Asteraceae Dum., Betulaceae A. Gray., Caprifoliaceae Juss., Clethraceae Klotzsch, Cornaceae (Dumort.) Dumort., Euphorbiaceae Juss., Ericaceae Juss., Oleaceae Hoffmanns. & Link, Polygalaceae R. Br. in Flinders, Primulaceae Vent., Rosaceae Juss., Rubiaceae Juss., Rutaceae Juss., Schisandraceae Blume, Solanaceae Juss., Theaceae D. Don, Violaceae Batsch., and Vitaceae Juss., with it being most common in the Ericaceae Juss. and Rosaceae Juss. families.

The researchers have identified gaultherin in various plant organs, including the aerial parts (such as flowers, leaves, stems, and fruits) and the underground parts (such as rhizomes and roots). Table 1 provides a comprehensive list of plant species in which gaultherin was detected, organised alphabetically by botanical family. It also includes the plant parts and extract types used in the study, the gaultherin content (if determined), and the qualitative and quantitative analytical methods employed.

Reliable literature reports on the content of gaultherin in plant substances are scarce (Table 1). Nevertheless, the most significant amount of this compound was determined for *Betula lenta* L. bark (3 mg/g fw) [24,25,35], *Filipendula ulmaria* (L.) Maxim. herb (2.75 mg/g dw) and flowers (2.78 mg/g dw) [36], and for *Gaultheria procumbens* L. leaves (22 mg/g fw), stems (7.65 mg/g fw), and rhizomes (3.35 mg/g fw) [23,31]. Relatively high levels of gaultherin were also reported for *Gaultheria procumbens* L. fruit (2.73–98.25 mg/g) [37], aerial parts (21.37–127.69 mg/g) [38], stem (10.52–185.98 mg/g dw) [38,39], and leaf (98.41–288.13 mg/g) [38,40] dry extracts. In turn, low contents, calculated per dry weight of the plant material, were recorded for fresh (0.04–0.45 mg/g dw) and dried (0.10–0.45 mg/g dw) leaves of *Camellia sinensis* (L.) Kuntze different cultivars [41,42,43].

**Table 1:** The occurrence of gaultherin in the plant kingdom.

Family	Species	Plant Part	Identification Method	Extract (Gaultherin Content If Determined)	References
Apocynaceae Juss.	*Poacynum hendersonii* (Hook.fil.) Woodson	flowers	UV, IR, FAB-MS, ^1^H NMR, ^13^C NMR	ethyl acetate–water (1:1, *v*/*v*) fraction of methanolic extract	[44]
Asteraceae Dum.	*Artemisia copa* Phil.	stems and leaves	UHPLC-Orbitrap-MS	infusion prepared with deionised water	[45]
Betulaceae A. Gray.	*Betula lenta* L.	bark	—	—	[22]
		bark	melting point, [α]_D_^30^, hydrolysis	3 g/kg fw of the bark	[24,25,35]
	*Ostryopsis davidiana* Decaisne.	bark	melting point, hydrolysis	–	[46]
Caprifoliaceae Juss.	*Lonicera caerulea* L.	fruits	UPLC-ESI-MS/MS	–	[47]
Clethraceae Klotzsch	*Clethra barbinervis* Siebold & Zucc.	stems	acid hydrolysis, HR-FAB-MS, ^1^H NMR; ^13^C NMR, 2D NMR	Acetone–water (4:1, *v*/*v*)	[48]
Cornaceae (Dumort.) Dumort.)	*Alangium alpinum* (C.B.Clarke) W.W.Sm. & Cave	whole plant (stems)	HR-ESI-MS, IR, CD, ^1^H NMR; ^13^C NMR, 2D NMR	ethanol–water (95:5, *v*/*v*) extract and its petroleum ether, ethyl acetate, and *n*-butanol fractions	[49]
Ericaceae Juss.	*Gaultheria leucocarpa* var. *cumingiana* (S.Vidal) T.Z. Hsu	–	melting point, [α]_D_^30^, hydrolysis	methanolic extract	[50]
	*Gaultheria fragrantissima* Wall.	stems and leaves	^1^H NMR, ^13^C NMR	methanol–water (70:20, *v*/*v*)	[51]
	*Gaultheria miqueliana* Takeda	leaves	melting point, [α]_D_^20^, hydrolysis	methanol–water (50:50, *v*/*v*)	[52]
	*Gaultheria procumbens* L.	leaves	melting point, UV, TLC	–	[53]
			LC-DAD-MS	26.00 mg/g fw of the leaves	[54,55]
			UHPLC-PDA-ESI-MS/MS, HPLC-PDA	liquid ME; gaultherin in mg/g dw of the leaves: 76.86 (April); 64.59 (May); 65.89 (June); 77.20 (July); 88.31 (August); 104.09 (September); 107.49 (October)	[56]
			melting point, UV, IR	–	[57,58,59]
			^1^H NMR, ^13^C NMR, 2D NMR, LC-MS/MS	water extract	[60]
			UHPLC-PDA-ESI-MS/MS, HPLC-PDA	ME; 98.41 mg/g dw of the extract	[38]
				content in mg/g dw of the extract: ME: 98.41; EAE: 288.13; BE: 127.81	[40]
		stems	UHPLC-PDA-ESI-MS^3^ HPLC-PDA	content in mg/g dw of the extract: ME: 93.76; EAE: 148.08; BE: 138.61; AE: 185.98; WE: 10.52	[39]
				ME: 93.76 mg/g dw of the extract	[38]
		fruits	HR-ESI-MS, ^1^H NMR, ^13^C NMR, 2D NMR; identification of aglycone (GC-MS) and sugars (acid hydrolysis, HPLC-PDA)	content in mg/g dw of the extract: ME: 48.89; EAE: 98.25; BE: 29.35; AE: 93.63; WE: 2.73	[37]
		aerial parts	UHPLC-PDA-ESI-MS/MS, HPLC-PDA	content in mg/g dw of the extract: ME: 96.51; MED: 75.68; DEF: 21.37; EAF: 30.25; BF: 127.69; WF: 27.81	[38]
		leaves, stems, and rhizomes	melting point, [α]_D_^20^, hydrolysis	leaves (22 g/kg); stems (7.65 g/kg); rhizomes (3.35 g/kg); leaves after enzymatic hydrolysis with boiling 60% alcohol (12.8 g/kg)	[23,31]
		leaves and fruits	UPLC-Q-TOF-HDMS	–	[60]
	*Gaultheria yunnanensis* (Franch.) Rehd.	leaves and stems	–	ethanol–water (95:5, *v*/*v*)	[15]
		aerial parts	–	ethanol–water (80:20, *v*/*v*)	[19]
		seeds	melting point, UV, IR, ^1^H NMR, ^13^C NMR, 2D NMR	ethanol–water (75:25, *v*/*v*)	[61]
		leaves and roots	UPLC-Q-TOF-HDMS	–	[60]
	*Gaultheria leucocarpa* var. *yunnanensis* (Franch.) T.Z. Hsu & R.C. Fang (Dianbaizhu)	aerial parts	MS, NMR	–	[62]
		–	isolation and identification by physicochemical properties and spectral analyses	–	[63]
		aerial parts	HPLC-Q-TOF-MS/MS HPLC-DAD	quantitative analysis of gaultherin after incubation with four intestinal segments (duodenum, jejunum, ileum, and colon)	[17,64]
		aerial parts	UPLC-LTQ-Orbitrap-MS/MS HPLC-DAD	the metabolism of gaultherin by human fecal microbiota in four intestinal segments (jejunum, ileum, cecum, and colon) and feces of rats in vitro by the HPLC-DAD method to methyl salicylate	[16]
	*Monotropa hypopitys* L.	–	melting point, [α]_D_^20^, hydrolysis	–	[10]
	*Monotropa uniflora* L.	whole plant	TLC, melting point, [α]_D_^23^, NMR	acetone extract	[65]
	*Vaccinium myrtillus* L.	leaves and fruits	–	–	[66]
Euphorbiaceae Juss.	*Euphorbia lathyris* L.	seeds	UPLC-QTOF-MS	ethanol–water-12 N HCl extract at pH = 2 (50:50:2, *v*/*v*)	[67]
Oleaceae Hoffmanns. & Link	*Olea europaea* L.	leaves	UPLC-ESI-TOF-MS	methanol–water (80:20, *v*/*v*)	[68]
Polygalaceae R. Br. In Flinders	*Securidaca longipedunculata* Fresen.	roots	hydrolysis	–	[69]
		roots	–	aqueous extract	[70]
		–	–	–	[71]
	*Polygala arillate* Buch.-Ham. Ex D.Don	stem bark	FAB-MS, ^1^H NMR, ^13^C NMR, 2D NMR	alcoholic extract	[72]
Primulaceae Vent.	*Primula officinalis* (L.) Hill	roots	melting point, fermentative hydrolysis	–	[30]
Rosaceae Juss.	*Prunus amygdalus* Batsch	seeds (almonds)	melting point, hydrolysis	the emulsion of almonds	[29]
	*Spiraea ulmaria*, *S. filipendula*, *S. gigantea* var. *rosea*	roots	melting point, [α]_D_^20^, hydrolysis	–	[28]
	*Filipendula glaberrima* Nakai	stems and roots	^1^H NMR, ^13^C NMR, FAB-MS	*n*-butanolic extract	[73,74]
	*Filipendula ulmaria* (L). Maxim.	flowers	melting point, [α]_D_^15^, hydrolysis	–	[75]
		flowers	–	alcoholic and water extracts	[76]
		herb and flowers	LC-DAD-ESI-MS/MS	methanolic extract from herb: 1.108 mg/g dw of the plant material (expressed in salicylic acid equivalents) and 2.756 mg/g dw of the plant material (expressed in salicin equivalents); methanolic extract from flowers: 1.098 mg/g dw of the plant material (expressed in salicylic acid equivalents) and 2.787 mg/g dw of the plant material (expressed in salicin equivalents)	[36]
		aerial parts	LC-PDA-MS	Methanol–40 mM ammonium formate buffer (aqueous) (20:80, *v*/*v*)	[77]
		aerial parts and roots	LC-DAD-MS/MS	methanolic extract	[78]
		aerial parts	UHPLC-HR-ESI-MS	methanol–water (80:20, *v*/*v*)	[79]
	*Filipendula vulgaris* Moench	aerial parts and roots	LC-DAD-MS/MS	methanolic extract	[80]
Rubiaceae Juss.	*Uncaria rhynchophylla* (Miq.) Jacks.	leaves and stems	HR-ESI-MS, NMR	ethanol–water (95:5, *v*/*v*)	[81]
Rutaceae Juss.	*Citrus grandis* L. Osbeck *cv. guanxiyou*	peels	UHPLC-TOF-MS	ethanol–water (80:20, *v*/*v*)	[82]
Solanaceae Juss.	*Physalis angulata* L.	stems and leaves	UV, CD, IR, HR-ESI-MS, ^1^H NMR, ^13^C NMR	ethanol–water (75:25, *v*/*v*) and its petroleum ether, ethyl acetate, and *n*-butanol fractions	[83]
Schisandraceae Blume	*Schisandra propinqua* (Wall) Hook. F. et Thoms.	seeds	UV, IR, ^1^H NMR, ^13^C NMR, MS	*n*-butanolic extract	[84]
Theaceae D. Don	*Camellia sinensis* var. *sinensis* cv. Maoxie	leaves	enzymatic hydrolysis, GC-MS	aqueous extract and its methanol–water (60:40, *v*/*v*) and methanolic fractions	[85]
	*Camellia sinensis* var. *sinensis* cv. Yabukita	leaves	HR-FAB-MS, ^1^H NMR, ^13^C NMR	aqueous extract and its methanol–water (20:80, *v*/*v*) fraction	[86]
	*Camellia sinensis* var. *sinensis* cv. Yabukita; *Camellia sinensis* var. *sinensis* cv. Chin-Shin-Oolong; *Camellia sinensis* var. *sinensis* cv. Benihomare	leaves	GC-MS	dried fresh leaves of three tea cultivars: Yabukita (18.2 mg/100 g of dried leaves), Chin-Shin-Oolong (10.0 mg/100 g), Benihomare (45.2 mg/100 g)	[87]
	*Camellia sinensis* var. *sinensis* cv. Yabukita	leaves	enzymatic hydrolysis, GC-MS	acetone powder from fresh tea leaves	[88,89]
	*Camellia sinensis* var. *sinensis* cv. Benihomare	leaves	GC-MS	dried fresh leaves (45.2 mg/100 g of dried leaves), withered leaves (40.9 mg/100 g), rolled leaves (13.7 mg/100 g), fermented leaves (0.8 mg/100 g)	[41]
	*Camellia sinensis* var. *sinensis* cv. Chin-Shin-Oolong	leaves	GC-MS	dried fresh leaves (9.3 mg/100 g of dried leaves), solar-withered leaves (10.6 mg/100 g), indoor-withered leaves (13.9 mg/100 g), oolong tea (17.0 mg/100 g)	[42,43]
	*Camellia sinensis* var. *sinensis* cv. Chinhsuan-Oolong	leaves	GC-MS	dried fresh leaves (4.4 mg/100 g of dried leaves), solar-withered leaves (3.9 mg/100 g), indoor-withered leaves (4.3 mg/100 g), oolong tea (6.8 mg/100 g)	
	*Camellia sinensis* var. *sinensis* cv. Zhuye	leaves	GC, HPLC-MS	alcoholic extracts	[90]
Violaceae Batsch.	*Viola cornuta* L., *V. tricolor* L.	flowers	UPLC-Q-TOF-HDMS	vine	[60]
Vitaceae Juss.	*Vitis* sp.	Verdichio, Trebbiano di Soave, Trebbiano di Lugana vines	UPLC-Q-TOF-HDMS	2.5–181.0 μg/L of vine (expressed in gaultherin equivalents)	[60]
Other	*Cinnamomum ramulus, Ilex pubescens, Sargassum*	Cinnamon twigs, Pubescent holly root, Seaweed	UPLC-MS/MS	–	[91]

ME: methanol–water dry extract (75:25, *v*/*v*; direct extraction); EAE: ethyl acetate dry extract (direct extraction); BE: *n*-butanol dry extract (direct extraction); AE: acetone dry extract (direct extraction); WE: water dry extract (direct extraction); dw: dry weight; fw: fresh weight.

## 3. Isolation Methods from Plant Materials

The challenge in isolating gaultherin from plant material lies in its rapid hydrolysis during extraction, which occurs when plant tissue is damaged, breaking it down into methyl salicylate and disaccharide primverose. This reaction is driven by a hydrolase-type enzyme in plant cells known as gaultherase [32,55]. Therefore, to reduce the hydrolysis of gaultherin, an extraction method must be designed to operate under conditions that inhibit or deactivate gaultherase action. So far, the researchers have proposed four approaches for isolating gaultherin from the plant material (Table 2).

The first report, dating back to 1928, concerns the extraction of gaultherin from the whole plant (likely aerial parts) of *Gaultheria procumbens* L. [23]. In the first step, Bridel and Grillon boiled the fresh plant material in water with added calcium carbonate, then crushed and re-extracted with water, which inhibited the hydrolytic activity of the gaultherase enzyme. The scientists combined the aqueous extracts and further distilled them under reduced pressure. The resulting residue was then extracted three times with a boiling ethanol–water mixture (90:10, *v*/*v*). Next, the alcoholic extracts were concentrated and treated with boiling ethyl acetate containing 5% ethanol, resulting in the crystallisation of gaultherin. The researchers purified the compound by recrystallisation in acetone with a small amount of water. In this way, the authors successfully obtained pure gaultherin with an isolation yield of 4.8 mg/g of fresh weight (fw) of the plant material.

The second technique was developed and patented in 2002 by an American research team [54]. Ribnicky and co-workers used fresh or fresh-frozen aerial parts of *Gaultheria procumbens* L., which the scientists first damaged in the presence of 70–95% ethyl alcohol and without the addition of a drying agent, thereby decreasing the risk of gaultherin hydrolysis by gaultherase. In the next step, the alcoholic extract was filtered or centrifuged to remove the solid matter, concentrated to dryness (in vacuo), and then dissolved in water or buffer (the authors have repeated this step once) to obtain a more purified form of gaultherin-rich fraction, from which pure gaultherin can be isolated with a yield of 5–25 mg/g fw of the plant material (preferably, the extract comprises at least 10 mg of gaultherin per gram fresh weight of the plant material). The advantage of this method over the previous one, developed over 70 years ago by Bridel and Grillon, is the use of fresh or fresh-frozen plant material and ethyl alcohol as an extractant, which allows the inhibition of the hydrolytic activity of the gaultherase enzyme. However, the disadvantage of this procedure is that gaultherin was obtained not in its pure crystalline form, but as a solid residue of a salicylate-rich fraction, containing only 12–18% gaultherin, as determined by HPLC.

The method developed by the Polish team of researchers in 2020 made it possible, unlike the procedure patented by Ribnicky, to obtain pure gaultherin, rather than a fraction rich in salicylates [37]. Michel and others used *Gaultheria procumbens* L. fruits in the extraction process and *n*-butanol as an extractant, allowing the elimination of the aqueous environment and, thus, the hydrolysis of gaultherin. The authors have utilised during the process, reflux extraction and high-performance preparative HPLC chromatography, which allowed for the reduction in the number of analysis steps and the isolation of pure gaultherin with an isolation yield of 4.3 mg/g fw of the plant material, similar to that reported by Bridel and Grillon [23].

**Table 2:** Isolation of gaultherin from plant material.

Plant Material	Extraction and Isolation Method	Isolation Process Description	References
*Gaultheria procumbens* L.—whole plant (leaves, stems, or rhizomes)	liquid extraction and crystallisation of gaultherin	Plant material: fresh whole plant (likely aerial parts); Immersion of the plant material in three times its weight of boiling water containing calcium carbonate, and boiled for 20 min; After cooling, the plant material is crushed and extracted again with fresh water; Distillation of the combined aqueous extracts under reduced pressure to remove volatiles; Three times extraction of the residue from the aqueous extract with boiling ethanol–water (90:10, *v*/*v*); Combination of the alcoholic solutions and evaporation to dryness under reduced pressure; Treatment of the resulting residue with boiling ethyl acetate containing 5% ethanol; Crystallisation of gaultherin during the first four treatments as prismatic crystals; Purification of the crude crystals by recrystallisation in acetone containing 0.8% water; Isolation yield of pure gaultherin: 4.8 mg/g fw of the plant material.	[23]
*Gaultheria procumbens* L.—aerial parts	subsequent liquid extraction and concentration (in vacuo) of the ethanolic extract to obtain a solid residue containing 12–18% (by weight, determined by HPLC) of gaultherin	Plant material: fresh or fresh-frozen aerial parts or, optionally, frozen tissues in liquid nitrogen; Grinding or otherwise disrupting (e.g., using a Polytron or a Waring blender) the plant tissue under solvent condition (preferably 70–95% ethanol or other solvent lacking a compound or structure removing water—a drying agent such as tragacanth, gelatine, a water-free sodium sulphate, a water-free magnesium sulphate, a water-free calcium chloride, a molecular sieve, membranes or combinations thereof, as examples of compounds and structures that bind, absorb, adsorb, or capture water molecules). The frozen, macerated tissue can be stored frozen for further processing; Removal of solid matter from the alcoholic extract (by filtration or centrifugation); Subjection of the alcoholic extract to an agent that removes the solvent component to produce a solid (powdered) gaultherin-containing residue (distilling the solvent on a rotary evaporator); Resuspension of the resulting dry alcoholic extract in water or buffer; Repetition of steps 4 and 5 to obtain a more purified form of gaultherin after removing any water-insoluble material (by centrifugation); Isolation yield of salicylate-rich fraction containing 12–18% of gaultherin; isolation yield of pure gaultherin: 5–25 mg/g fw of the plant material.	[54]
*Gaultheria procumbens* L.—fruits	refluxed extraction and HPLC-preparative isolation of gaultherin	Plant material: dried fruits; Refluxed extraction of the plant material (100 g) with *n*-butanol (3 times, 300 mL × 2 h each time); Evaporation of the liquid *n*-butanolic extract at 40 °C (in vacuo) to give dry *n*-butanolic extract; Preparative HPLC-PDA isolation of gaultherin from dry *n*-butanolic extract using an LC-20AP system equipped with a preparative pump, PDA detector, thermostated autosampler and column oven, and an XB-C18 Kinetex column (5 μm, 150 mm × 22.1 mm i.d.). A linear gradient system was applied for preparative elution with the following profile: 0–25 min, 2–20% B (*v*/*v*), 25–30 min, 20–2% B (*v*/*v*), where the mobile phase (A) was water-formic acid (100: 0.1, *v*/*v*), and (B) was acetonitrile-formic acid (100: 0.1, *v*/*v*). Separation was performed at 25 °C, with a flow rate of 20 mL/min and an injection volume of 200 μL. Before the isolation, samples of dry *n*-butanolic extract were dissolved in DMSO (4 mL) and filtered through a PTFE syringe filter. The fraction collection was triggered automatically by a signal from the UV-Vis detector at λ = 285 nm (methyl salicylate glycosides); Isolation yield of pure gaultherin: 27.9 mg/g dw of the extract (10.8 mg/g dw of the plant material; 4.3 mg/g fw of the plant material).	[37]
*Gaultheria fragrantissima* Wall. (synonymous name: Gandapura)—leaves	UV photo extraction and isolation of a gaultherin-rich fraction	Plant material: fresh-frozen leaves; Extraction of the plant material with ethanol solvent (ethanol concentrations at 85, 90, and 96%) in UV-photo-extractor (extraction times: 10, 30, and 50 min; solvent: feed ratio is 5:1; pH maintained at pH 4.8 using a pH buffer; the stirrer rotational speed and chopper blade rotation are 75 and 125 rpm, respectively; a drying agent is calcium chloride); Separation of the solids from the extract by filtration or centrifugation; One-stage osmotic dehydration by the addition of chemicals or heating to remove the solvent; Resuspension of the heated extract in buffer solution or water to obtain the gaultherin-rich fraction; Conclusions: the higher the extraction time, the higher the yield of gaultherin; however, the increase in the ethanol concentration up to 96% resulted in a decrease in gaultherin from the 90% ethanol concentration; Percentage content of gaultherin in the salicylate-rich fraction (the optimal conditions: ethanol 90% and extraction time 50 min): 22.8%.	[92,93]

dw: dry weight; fw: fresh weight.

The method successfully inhibited gaultherase hydrolytic activity. It also efficiently extracted the compound from the plant material and then isolated it in the pure form from the *G. procumbens* fruit dry extract.

The latest procedure proposed by the Indonesian team of researchers, unfortunately, only allowed for the obtaining of a salicylate-rich fraction containing 22.8% gaultherin, rather than the pure compound [92,93]. The novelty lay in using a photo extractor-UV machine and leaves of *Gaultheria yunnanensis* (Franch.). Rehd. (synonymous name, Gandapura) as plant material. Similarly, Sutrisno and others [93] used different percentages of ethanol and water mixtures in the extraction process to deactivate the gaultherase enzyme and draw the gaultherin-rich fraction. This study selected 90% ethanol and an extraction time of 50 min as the optimal conditions.

Scientists are obtaining commercially available gaultherin, likely from various *Gaultheria* species; however, detailed information is unavailable due to patent protection. At the same time, its availability on the market as a commercial standard is limited mainly to Asian countries, including China. Additionally, 20 mg of the compound costs between USD 130 and USD 1200, depending on the manufacturer. The high price of this methyl salicylate glycoside is a significant restriction in broader studies of gaultherin and the reason for the lack of clinical trials. Therefore, the development of further rapid and cost-effective methods for obtaining this compound in its crystalline form with a purity of at least 95% (as determined by HPLC) should become the goal of extended phytochemical studies.

In addition to isolation from plant sources, the researchers have demonstrated the feasibility of large-scale synthesis of gaultherin through an efficient phase-transfer catalysis approach. Wang and co-workers [34] reported a practical synthetic route that uses methyl salicylate as the aglycone and commercially available D-glucose and D-xylose to assemble the disaccharide moiety under mild conditions. The method eliminates the need for anhydrous or strictly inert atmospheres by employing tetrabutylammonium bromide as a phase-transfer catalyst, thereby significantly simplifying the procedure for potential industrial applications. The overall process is amenable to scale-up and yields gaultherin with good efficiency and stereoselectivity. This synthetic strategy provides a reliable alternative to plant extraction, making it a viable option for pharmaceutical development and commercial-scale production [34].

## 4. Pharmacokinetic Properties

### 4.1. Absorption, Distribution, Metabolism, and Excretion Based on the ADMETlab 3.0 Web Server

The pharmacokinetic profile of gaultherin was assessed using ADMETlab 3.0, a widely utilised web-based tool that predicts pharmacokinetic (absorption, distribution, metabolism, and excretion) and toxicity characteristics based on chemical structure [94], supporting the advancement of drug discovery and design [95]. However, the database predictions may not fully capture the complex in vivo interactions and metabolic changes in a chemical compound in the human body, or rare adverse effects, which limit their applicability in specific advanced pharmacological assessments.

The intestinal absorption of gaultherin was estimated by calculating the predicted permeability level of Caco-2 (human colon adenocarcinoma) and MDCK (Madin–Darby canine kidney) cells, as well as using the PAMPA (parallel artificial membrane permeability) assay. The results of Caco-2 permeability (−5.58 log cm/s; optimal permeability: >−5.15 log cm/s), MDCK permeability (−4.995; high passive permeability: >20 × 10^−6^ cm/s), and PAMPA assay (0.99 log P_eff_; low permeability: <2.0 log P_eff_) suggested poor passive diffusion absorption of orally administrated gaultherin in the gastrointestinal tract. The probability that the compound functions as an inhibitor of a P-glycoprotein (P-gp), a highly versatile efflux transporter and member of the ATP-binding cassette protein family, was low (0.0; optimal range for excellent P-gp inhibitors: 0.7–1.0), with high potential of being a substrate of P-gp (0.721; optimal range for excellent P-gp substrates: 0.7–1.0). Weak P-glycoprotein inhibitors that are also strong P-gp substrates can provide self-limiting efflux effects, potently triggering P-gp transport only when concentrations are low, which helps avoid systemic drug–drug interactions and toxicity [96]. Meanwhile, being strong substrates supports targeted efflux in tissues overexpressing P-gp, such as the intestine, blood–brain barrier, or cancer cells, enabling site-selective pharmacokinetics and potentially minimising off-target exposure [97]. At the same time, the probability of human intestinal absorption, expressed as HIA (0.241; optimal range: 0.7–1.0), as well as the F_30%_ and F_50%_ oral bioavailability indexes (0.88 and 0.76, respectively; optimal range: 0–0.3), were defined as poor. Only the F_20%_ bioavailability (0.04; optimal range: 0–0.3) index was estimated as excellent. The human oral bioavailability of methyl salicylate glycosides, including gaultherin, has not been directly measured in clinical studies. However, this parameter for gaultherin was predicted to range from 20% to 30%, suggesting effective absorption similar to that observed for other methyl salicylate glycosides tested in animal models [6,98,99].

The second step of ADME analysis involved evaluating the distribution characteristics. The estimated plasma protein binding for gaultherin was 55.39%, which falls below the ideal threshold of 90% for drug candidates. This value indicates a moderate binding affinity to plasma proteins, potentially allowing for a stable therapeutic index that is less susceptible to variations in drug concentration. Furthermore, the unbound fraction of gaultherin in serum was estimated at 49.02%, exceeding 20%, and thus considered relatively high, which can contribute to the effective therapeutic action of the compound at lower doses. The ADMETlab 3.0 predicted the volume of distribution (VDss) to be −0.59 L/kg, which can be interpreted as a poor tissue distribution (optimal range: 0.04–20 L/kg). However, the expected negative VDss value for gaultherin likely results from a modelling error, yet suggests that the compound remains primarily in plasma and does not distribute significantly to tissues. The predicted possibility of gaultherin crossing the blood–brain barrier was low (0.057), suggesting minimal risk of central nervous system-related side effects. Lastly, gaultherin presented a high chance of being an inhibitor of the organic anion-transporting polypeptides OATP1B1 (0.998) and OATP1B3 (0.999)—necessary hepatic uptake transporters and multidrug resistance protein 1, MRP1 (0.779)—an integral transmembrane efflux transporter, which could lead to drug–drug interactions. At the same time, the compound showed a low probability of being a breast cancer resistance protein BCRP inhibitor (0.061)—a vital ATP-binding cassette efflux transporter.

The metabolism parameters indicated that gaultherin had no inhibitory effects on key human cytochrome P450 enzymes, including CYP1A2, CYP2C19, CYP2C9, CYP2D6, CYP3A4, and CYP2B6 isoforms. The compounds that do not inhibit the major cytochrome P450 enzymes offer several significant benefits for potential drug development, such as a good safety profile, predictability, and better clinical suitability, especially in patients taking multiple medications, which also minimises interaction risks and supports a wider therapeutic application. From the analysed enzymes, gaultherin may inhibit CYP2C8 (probability at 0.885 level). Additionally, the human liver microsomal stability of gaultherin was estimated to be high (0.004), contributing to a longer duration of action, improved bioavailability, fewer side effects, and a greater likelihood of clinical success.

Regarding excretion, two primary parameters were evaluated. First, the drug clearance rate (CL_plasma_), defined as the volume of plasma from which the drug is completely removed per unit of time, was calculated at 1.45 mL/min/kg, which is considered low (values below 5 mL/min/kg). Additionally, the predicted half-life time (T_1/2_) of gaultherin was relatively short, at approximately 3.57 h, indicating quick removal from the human body. This rapid elimination can enhance safety, allow for greater dosing flexibility, and facilitate better clinical control, particularly for drugs that require precise therapeutic management or carry a risk of side effects. Although more frequent dosing may be necessary compared to long-acting drugs, this profile supports a safer and more versatile treatment option. All these factors should be carefully considered when determining an appropriate dosing regimen to balance efficacy with minimal adverse effects.

### 4.2. Bioavailability Assessment Based on Experimental Studies

Gaultherin is a precursor compound that becomes pharmacologically active after metabolic transformation within the human body. The oral bioavailability and other pharmacokinetic parameters of methyl salicylate glycosides, including gaultherin, have not been directly measured in humans in clinical studies. Available data come mainly from in vivo studies in animal models, including mice, rats, dogs, and rhesus monkeys. The most important information regarding the pharmacokinetic parameters of gaultherin is presented in Table 3.

Upon oral administration, gaultherin is broken down into methyl salicylate, mainly by β-glycosidase enzymes from intestinal microbiota (Figure 1). The presence of a terminal xylosyl group in the chemical structure of this compound slows down its metabolism because xylosyl groups are more resistant to hydrolysis by gut microbiota than glucosyl groups, which significantly affects the metabolic rate and pathway of gaultherin [16]. The liberated methyl salicylate is absorbed through the gastrointestinal mucosa or skin (in topical applications). Further enzymatic action by nonspecific esterases in the intestine, blood, and liver facilitates the release of salicylic acid, the agent responsible for its therapeutic effects [15,17,18]. Salicylic acid is next distributed systemically and binds to plasma proteins. After reaching the liver, it undergoes conjugation, primarily forming salicyluric acid, salicyl phenolic glucuronide, and salicyl acyl glucuronide, which are ultimately excreted by the kidneys [100].

Salicylic acid, the active metabolite of gaultherin, targets sites of inflammation and inhibits the COX-2 enzyme after entering the bloodstream, thereby decreasing the production and release of pro-inflammatory mediators [15]. Since prostaglandins mediate pain, inflammation, and fever, their suppression leads to symptom relief. Importantly, this metabolic route enables gaultherin to provide anti-inflammatory benefits without the gastric irritation commonly associated with aspirin due to the lack of inhibition of the COX-1 isoform [15,101].

Zhang and associates [15] demonstrated that gaultherin is not detected in plasma after oral administration to the animals (mice and rats), confirming it is not absorbed intact but metabolised in the gut, i.e., hydrolysed by β-glycosidase enzymes from intestinal bacteria, producing methyl salicylate, which is further metabolised by esterases present in the intestine, blood, and liver, ultimately yielding salicylate. The authors also demonstrated that the metabolites of gaultherin appear in animal plasma several hours after administration (T_max_: ~5 h in mice and 7.5 h in rats). The slow release of salicylate ensures sustained plasma concentrations, avoiding high peak levels that might trigger side effects [15].

**Table 3:** Differences in pharmacokinetics and pharmacodynamics between synthetic aspirin and natural gaultherin.

Parameter	Aspirin (Acetylsalicylic Acid)—Synthetic NSAID	Gaultherin and Other Methyl Salicylate Glycosides	References
Absorption	Rapid, absorbed mainly in the stomach and upper small intestine (duodenum).	Slow, intestinal absorption (requires bacterial and esterase metabolism).	[6,15,18,100,102,103,104,105]
Bioavailability	High and predictable.	Variable, dependent on gut flora and enzyme activity.	[6,15,18,103,104,106]
First-pass metabolism	Undergoes significant hepatic first-pass metabolism.	Minimal, as activation occurs before absorption.	[6,15,18,100,102]
Elimination	Renal, primarily as salicylate metabolites.	Renal, post-conversion to salicylate.	[6,15,100,102,104,105]
Parent compound in plasma	Detected; rapidly absorbed after oral administration.	Not detectable; it acts as a prodrug.	[6,15,106]
Primary active metabolite	Salicylate, directly from aspirin hydrolysis.	Salicylate, via methyl salicylate intermediate.	[15,100]
Time to peak plasma level (T_max_)	~0.5–1 h (both animals and humans).	~5 h (mouse), ~7.5 h (rat).	[15,18,106]
Peak plasma concentration (c_max_)	Rapid rise; variable, depending on the dose and formulation.	~60 μg/mL (mouse), ~70 μg/mL (rat).	[6,18,106]
Duration of salicylate exposure	Shorter due to rapid peak and decline.	Prolonged due to slow conversion.	[15,18,100]
Half-life (T_1/2_)	Aspirin: ~15–20 min (plasma); Salicylate: 2–3 h (dose-dependent).	Salicylate formed from gaultherin has an extended half-life (~4–6 h, depending on formulation).	[15,18,102,103,105,106]
Stomach	As an organic acid, aspirin is quickly released and induces tissue injury after oral administration. Only a small amount of acetylsalicylic acid ionises in the stomach due to its weak acidity, allowing it to be rapidly absorbed through the gut lining under acidic conditions by passive diffusion.	Gaultherin passes through the stomach without being degraded due to the presence of an ester group (blocked acid group), which can significantly reduce gastrointestinal irritation.	[5,6,15,16,18,100,104]
Intestine	In the small intestine, aspirin is absorbed more slowly due to the higher pH and expanded surface area, which promote greater ionisation. During an overdose, the drug’s absorption is further delayed by the formation of concretions.	The intestinal bacteria produce β-glycosidases, which break down the ester linkage of gaultherin, producing methyl salicylate.	[5,6,15,16,18,100,104]
COX-1 inhibition	Aspirin induces irreversible inhibition of gastrointestinal COX-1, which can reduce the risk of heart attack and stroke. At the same time, this activity results in the loss of the cytoprotective effect of prostaglandin E2 and prostaglandin I2 on the gastric mucosa (significant ulcerogenesis). This impact causes several adverse side effects, especially gastric mucosa injury, gastric ulcer, gastric bleeding, and dyspepsia.	Negligible impact on COX-1 activity.	[100,104,107,108]
COX-2 inhibition	Aspirin is a non-selective, irreversible COX-2 inhibitor.	Gaultherin and other methyl salicylate glycosides are selective COX-2 inhibitors.	[5,100,104,108]
Platelet aggregation	Aspirin strongly and irreversibly inhibits platelet aggregation, causing serious side effects such as bleeding, ulcers, or intracranial bleeding.	Negligible impact on platelet aggregation (no irreversible inhibition of platelets).	[6,100,104,109]
Therapeutic dosage	In vivo studies conducted in animal models have shown that: aspirin (dose 200 mg/kg) and gaultherin (dose 400 mg/kg) reduced the ear swelling after application of croton oil and the visceral pain induced by acetic acid to a similar extent;		[15]
	aspirin (dose 200 mg/kg) and a salicylate-rich fraction (dose 800 mg/kg) reduced the ear oedema with topical application of croton oil, the number of writhing and stretching induced by the acetic acid, and the time of paw licking induced by formalin at a similar level.		[19]
Therapeutic implication	Aspirin is suitable for acute relief but is limited by gastrointestinal and bleeding risks.	Gaultherin has the potential for safer long-term use due to extended release and minimal gastrointestinal risk.	[15,16,18,19,100,103,104]
Undesirable effects	Aspirin can cause stomach pain, heartburn, nausea, and vomiting, and increase the risk of ulcers and bleeding. It can also contribute to cardiovascular events, including increased blood pressure and a heart attack. Allergic reactions, kidney problems, and tinnitus (ringing in the ears) are no less important.	Gaultherin may cause allergic skin reactions. Mild side effects, including nausea, vomiting, rash, dizziness, and breathing difficulties, have also been reported.	[15,104,107,110]

NSAID: nonsteroidal anti-inflammatory drug; COX: cyclooxygenase.

In the research on the metabolism of gaultherin, MSTG-A, and MSTG-B by gut microbiota, including human faecal microbiota and microbiota in different intestinal segments (jejunum, ileum, cecum, and colon) and faeces of rats, Dong and co-workers [16] demonstrated that caecal microbiota had the strongest ability to metabolise gaultherin to methyl salicylate. The authors have also shown that faecal and colonic microbiota were partially effective but less so than caecal microbiota. At the same time, jejunal and ileal microbiota exhibited limited or no capacity to hydrolyse gaultherin. In addition, the authors reported the presence of methyl salicylate, the primary active metabolite of gaultherin and other methyl salicylate glycosides, after 2–4 h of incubation with gut microbiota [16].

Wang and others [17] conducted an in-depth study of the intestinal metabolism and absorption mechanisms of multiple components in *Gaultheria leucocarpa* var. *yunnanensis* (Franch.). T.Z. Hsu & R.C. Fang (Dianbaizhu), including gaultherin, MSTG-A, and MSTG-B, particularly focusing on the anti-rheumatic arthritis fraction. In studies conducted on both normal and rheumatoid arthritis model rats, the authors showed that gaultherin and two other methyl salicylate triglycosides are present in significantly higher amounts in the small intestine (especially duodenum and ileum) than in the colon. Additionally, all three salicylates exhibited good intestinal permeability (Peff > 0.5 × 10^−4^ cm/s; Fa > 40%) and were identified as P-gp substrates, suggesting that efflux transport may limit their absorption [17].

Further bioactivity and metabolic targeting research by Wang and co-workers [111] demonstrated strong binding affinity (−104.91 kcal/mol) of gaultherin with ATP2B2 (also known as Plasma Membrane Calcium ATPase; PMCA2), a plasma membrane Ca^2+^ transporter, that plays a crucial role in maintaining calcium homeostasis in cells. Molecular docking and dynamics simulations confirmed that the gaultherin-ATP2B2 complex is highly stable, suggesting a robust and sustained interaction. In addition, this study indicated that gaultherin might exert therapeutic effects by modulating calcium homeostasis, ion channel function, and synaptic signalling. These mechanisms support its potential role not only in anti-inflammatory applications but also in neurological conditions such as schizophrenia [111].

The latest study by Wang and others [18] in a model of rheumatoid arthritis rats confirmed the previous findings of Zhang and associates [15] that gaultherin is not directly absorbed into the bloodstream; instead, its metabolites (especially salicylate) appear in plasma, showing slow-release kinetics with delayed T_max_ (~5–7.5 h), ensuring sustained exposure and reduced toxicity. The authors also demonstrated that gaultherin and its metabolites distribute widely in tissues, particularly in the kidney, liver, lung, and spleen; the last organ exhibited the highest accumulation of prototypical compounds (gaultherin, MSTG-A, and MSTG-B) and metabolites, suggesting it may be a key target organ for its anti-inflammatory effects. The work also showed that more than twenty different metabolites of prototypical methyl salicylate glycosides, identified by LC-MS, were formed by transformation via hydrolysis, hydroxylation, sulfation, methylation, and glucuronidation. These metabolites are considered to be more pharmacologically active than the parent compound in animal models of rheumatoid arthritis [18].

The researchers also conducted pharmacokinetic studies in animal models, i.e., dogs and rhesus monkeys, for other representatives of methyl salicylate glycosides, i.e., methyl salicylate 2-*O*-β-D-lactoside (MSL) [98,99]. Zhang and co-workers [99] conducted in vivo studies on beagle dogs, demonstrating that orally administered MSL was not detected in plasma. Instead, salicylic acid, the primary metabolite, was rapidly detected within 0.25 h, peaking 8–10 h post-administration. This effect suggested that MSL was hydrolysed by intestinal microbiota or enzymes in the gastrointestinal tract before or during absorption. The authors also examined the route-dependent metabolism of MSL. Zhang and others [99] found that after oral administration, only salicylic acid appeared in the dog’s plasma. On the other hand, only MSL was detected after intravenous administration, confirming that MSL is metabolised before absorption when taken orally. In pharmacokinetic studies, the half-life (T_1/2_) of salicylic acid derived from MSL increased with dose (from ~2.3 h to ~5.6 h), and the absolute oral bioavailability of MSL was high (86.5–90.9%) based on salicylic acid plasma levels. The results confirmed that, like gaultherin, MSL acts as a prodrug, releasing the active compound salicylic acid in the intestine, which allows it to avoid gastric irritation, a common issue with aspirin, making MSL a safer anti-inflammatory option [99].

Further pharmacokinetic studies of MSL, conducted by He and others [98] in a primate model, showed that after oral administration to rhesus monkeys, MSL was not detected in plasma; only salicylic acid was present, confirming that MSL is hydrolysed before absorption. Conversely, following intravenous administration, only MSL was found in plasma, with no trace of salicylic acid, reinforcing that the metabolic transformation occurred pre-systemically (in the gut). Pharmacokinetic characteristics confirmed that salicylic acid, the active metabolite of MSL, appeared rapidly in plasma (within 0.5 h), with a peak concentration occurring around 7 h. The author noted that the oral bioavailability of MSL exceeded 100%. Such findings suggested non-dose-proportional kinetics and prompted caution for clinical dosing. Compared to other experimental model animals, the metabolic pathway (oral conversion to salicylic acid, intravenous persistence of MSL) in rhesus monkeys mirrored results previously seen in dogs and rodents (mice and rats).

However, in both dogs and monkeys, the parent compound MSL was not detectable after oral administration; only its metabolite, salicylic acid, appeared in plasma, indicating extensive presystemic metabolism. In contrast, in rodents, MSL itself was detectable, suggesting less extensive first-pass metabolism compared to dogs and monkeys. MSL was also absorbed more rapidly in monkeys (shorter T_max_) than in dogs or rodents, suggesting faster gastrointestinal transit or uptake in primates. Additionally, rodents and dogs exhibited more predictable (linear) pharmacokinetics across the dose range. At the same time, monkeys exhibited poor dose proportionality, with non-linear increases in c_max_ and AUC, indicating saturation of metabolic or transport processes at higher doses [98,99].

While both compounds, i.e., gaultherin and MSL, serve as prodrugs of salicylic acid and follow similar metabolic fates, MSL generally offers more favourable pharmacokinetics, including faster hydrolysis, greater bioavailability, and more predictable absorption, when compared to gaultherin, whose pharmacokinetics are slower and more variable due to the difference in the structure of the sugar moiety and dependence on gut microbiota and enzymatic activity.

The therapeutic effects attributed to gaultherin are presumed to arise from its enzymatic conversion to salicylic acid, which is systemically available and pharmacologically active. While this prodrug mechanism has been demonstrated in preclinical models [15], the absence of direct human pharmacokinetic data represents a limitation. Therefore, while current findings suggest gaultherin may serve as a viable source of salicylic acid in vivo, definitive conclusions about its clinical efficacy must await confirmation through dedicated human bioavailability and pharmacokinetic studies.

Such studies would be critical to determine the extent and consistency of gaultherin’s metabolic conversion across individuals, as interindividual variability in gut microbiota composition and enzymatic activity could significantly impact its systemic exposure and therapeutic outcomes. Furthermore, identifying the primary sites and rates of hydrolysis in humans would aid in optimising formulation strategies or selecting appropriate patient populations. Until these data are available, extrapolation from animal models must be interpreted with caution, and gaultherin’s role should be viewed as complementary to that of better-characterised compounds, such as MSL, in the context of salicylate-based therapy.

## 5. Safety of Use

### 5.1. The Toxicology Study Based on the ADMETlab 3.0 Web Server

The potential toxicological profile of gaultherin was evaluated using the ADMETlab 3.0 web server, an online tool designed to predict compounds’ pharmacokinetic characteristics and toxicity risks based on their molecular structure [94]. The analysis indicated that gaultherin has a very low probability of causing liver, nervous system, blood, immune, or genetic toxicity, as well as low potential for being carcinogenic, cytotoxic (against A549 lung cancer and HEK293 kidney cells), or harmful to respiratory function. It also showed minimal risk of blocking the hERG potassium channel, a key factor in cardiac electrical activity. Predictions classified the compound as non-corrosive to the eyes, although it may have a moderate chance (0.373; medium eye corrosion/irritation potential: 0.3–0.7) of causing eye irritation. Oral toxicity in rats was deemed low, with a predicted value of 0.003 (low toxicity, >500 mg/kg). The estimated maximum recommended daily dose by the FDA for humans was rated as excellent, suggesting a very low risk of harm at exposures up to 0.009 mmol/kg/day. Despite a high Ames mutagenicity score (0.773), which implies a potential for genetic mutation in bacterial assays, such results often yield false positives and do not necessarily reflect human health hazards [112]. One area of concern was the compound’s high potential (0.98; high skin sensitisation: 0.7–1.0) for inducing skin sensitisation, which may limit its dermatological applications. Gaultherin also presented a medium probability of being nephrotoxic (0.613; medium nephrotoxicity: 0.3–0.7) and a high potential of being ototoxic (0.942; high ototoxicity: 0.7–1.0). The DILI (drug-induced liver injury) index was also categorised as medium (0.576; medium probability of DILI: 0.3–0.7).

Given these findings, the scientists should pay particular attention to the predicted nephrotoxicity and ototoxicity of gaultherin. Although these results are derived from computational models, they highlight the need for further preclinical evaluation. Both nephrotoxicity and ototoxicity are often dose-dependent phenomena, particularly when mediated by salicylate metabolism or prolonged systemic exposure [113,114]. Accordingly, controlled in vivo studies are warranted to determine whether gaultherin’s potential adverse effects manifest at therapeutic concentrations or only at suprapharmacological levels. Early-stage preclinical screening, including auditory brainstem response testing and renal histopathology, should be incorporated to assess these risks more accurately. Addressing these endpoints during the safety assessment phase is critical before considering clinical development.

### 5.2. The Cellular Safety Studies

The cellular biocompatibility of gaultherin has been confirmed so far in two in vitro cell-based models of human neutrophils and RAW264.7 murine macrophages. The general trend of the cellular safety of gaultherin on non-tumorigenic cells is consistent with the available reports on the cytocompatibility of other representatives of methyl or ethyl salicylate glycosides (Table 4).

## 6. Biological Activity

**Figure 2:**
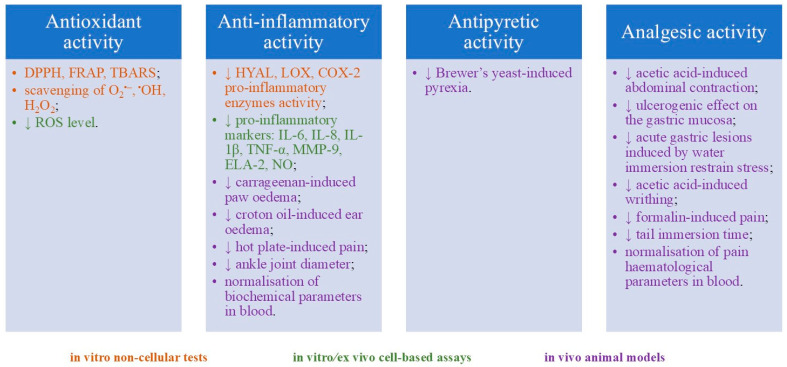
Summary of gaultherin biological activity. Abbreviations: DPPH: DPPH^•^ radical scavenging test; FRAP: antioxidant activity expressed in millimoles of Fe^2+^ ions/g of tested compound (Ferric Reducing Antioxidant Power); TBARS: linoleic acid oxidation inhibition test that measures the level of thiobarbituric acid reactive substances produced; O_2_^•−^: superoxide radical anion scavenging test; ^•^OH: hydroxyl radical scavenging test; H_2_O_2_: hydrogen peroxide reduction test; ROS: reactive oxygen species; HYAL: hyaluronidase inhibition assay; LOX: lipoxygenase inhibition assay; COX-2: cyclooxygenase-2 inhibition assay; IL: interleukin; TNF-α: tumour necrosis factor-α (tumour necrosis factor-α); MMP-9: matrix metalloproteinase-9; ELA-2: human elastase-2; NO: nitric oxide inhibition assay; (↓) downregulation.

The literature on the biological effects of gaultherin and gaultherin-rich plant fractions remains scarce (twelve articles, according to the databases included in this paper). This review provides a comprehensive overview of the anti-inflammatory, antipyretic, analgesic, and antioxidant activity, detailing the disease models used, study types and parameters, dosages tested, specific biological activities assessed, and key findings (Figure 2, Table 5). The discussion section focuses on synthesising the data, particularly the identified or proposed mechanisms of action, conclusions drawn from the research, and areas that warrant further investigation.

### 6.1. Anti-Inflammatory Activity

The anti-inflammatory activity is the leading biological effect of gaultherin, as suggested by traditional medicine regarding plants rich in this phytochemical [119]. Consequently, most literature data, including non-cellular and cell-based in vitro and ex vivo assays, as well as in vivo animal models, have been devoted to this topic. The researchers have demonstrated that gaultherin significantly inhibits the three key pro-inflammatory enzymes—namely, hyaluronidase, lipoxygenase, and cyclooxygenase-2—in in vitro cell-free assays (Table 5). Gaultherin showed the highest inhibitory activity towards cyclooxygenase-2 and hyaluronidase, and to a lesser extent to lipoxygenase, especially when compared to the positive controls of indomethacin, dexamethasone, quercetin, zileuton, celecoxib, and rosmarinic acid [20,37,38,39,48,119]. In addition, molecular docking of gaultherin (in silico assay) revealed a strong binding affinity in the active sites of COX-2 (−9.40 kcal/mol), exceeding the binding affinity of aspirin, which docked at −6.40 kcal/mol (COX-2) and −4.42 kcal/mol (COX-1) [20,120]. These differences highlight gaultherin’s higher selectivity and potential efficacy toward COX-2 over COX-1 at the molecular level, aligning with its observed biological anti-inflammatory effects. The docking studies further demonstrated that gaultherin engages in favourable hydrogen bonding and hydrophobic interactions within the active site residues, particularly in the enlarged COX-2 side pocket, which contributes to its selective inhibition profile.

Further research revealed that gaultherin at 25–75 μM markedly suppressed the release of key pro-inflammatory cytokines, i.e., IL-8, IL-1β, and TNF-α, as well as tissue-remodelling enzymes such as matrix metalloproteinase-9 and human neutrophil elastase-2 (ELA-2), in an ex vivo human neutrophils stimulated with bacterial lipopolysaccharide (LPS) or *N*-formyl-L-methionyl-L-leucyl-L-phenylalanine (*f*MLP) in the presence of cytochalasin B [37,38,39,119]. Among these effects, the most pronounced inhibition was observed in the secretion of IL-1β, ELA-2, and TNF-α. In addition, gaultherin (0.3–3.0 μg/mL) significantly downregulated the secretion of TNF-α, IL-1β, and IL-6 in LPS-stimulated RAW264.7 murine macrophages in vitro [115]. Another study indicated that gaultherin (0.3–3.0 μg/mL) substantially diminished the production of nitric oxide (NO) in LPS-stimulated RAW264.7 murine macrophages in vitro [34,83,115].

The potent anti-inflammatory activity of gaultherin has also been demonstrated in in vivo studies conducted in animal models, including mice and rats. Zhang and co-workers [15] showed that gaultherin (400 mg/kg), isolated from the stems and leaves of *Gaultheria yunnanensis* (Franch.) Rehd. significantly reduced ear swelling after application of croton oil to the inner surface of the right ear of mice. Zhang and colleagues [19] further demonstrated that a salicylate-rich fraction (200–800 mg/kg), comprising approximately 50% gaultherin and derived from the stems and leaves of *Gaultheria yunnanensis* (Franch.) Rehd., exhibited notable anti-inflammatory effects in animal models, significantly reducing ear oedema induced by croton oil in mice and paw swelling triggered by carrageenan in rats. Similarly, Alam and others [20] showed that the salicylate-rich fraction (100–300 mg/kg), this time obtained from stems and leaves of *Gaultheria trichophylla* Royle, presented significant anti-inflammatory (carrageenan- and croton oil-induced paw oedema models) effect in mice. A subsequent study conducted by the same team of scientists from Pakistan and Saudi Arabia demonstrated a significant reduction in joint inflammation by the salicylate-rich fraction in rats treated with Freund’s complete adjuvant [21].

### 6.2. Antipyretic Activity

Methyl salicylate glycosides, including gaultherin, inhibit NF-κB and MAPK signalling pathways, both of which regulate the expression of cytokines and COX-2 enzyme, thus contributing to downregulation of PGE2 production, a key mediator of fever [5,6].

To date, only one study conducted in an animal model has demonstrated the antipyretic activity of gaultherin-rich plant extract (Table 5). Alam and others [20] showed a significant antipyretic effect (Brewer’s yeast-induced pyrexia assay in mice) observed as a reduction in rectal temperature in the 5th hour of the experiment after oral administration of salicylate-rich fraction (100–300 mg/kg).

### 6.3. Analgesic Activity

Methyl salicylate glycosides, such as gaultherin, exhibit both anti-inflammatory and analgesic properties that are closely interconnected. Their anti-inflammatory activity contributes to their analgesic (pain-relieving) effects, as inflammation is a prominent source of chronic pain in many conditions [121]. Moreover, these glycosides offer a safer profile compared to free methyl salicylate, as they typically cause less gastric irritation, making them promising candidates for managing pain and inflammation simultaneously [6].

Gaultherin exhibited notable analgesic (antinociceptive) activity in a model of acetic acid-induced writhing test in mice [15]. The compound (400 mg/kg) significantly reduced abdominal contractions caused by acetic acid, though slightly less potently than an equimolar dose of aspirin (Table 5). At the same time, findings indicated that gaultherin, administered at 100 mM (equivalent to 330 mg/kg), did not produce any harmful effects on the gastric mucosa, as assessed by the mean lesion score, nor did it influence the development of stress-induced ulcers caused by water immersion restraint in rats. Further research by another team of Chinese researchers confirmed the relevant analgesic activity of a salicylate-rich fraction (200–800 mg/kg) on acetic acid-induced writhing, formalin-induced pain, and the hot plate test in mice [19]. Zhang and co-workers reported a dose-dependent reduction in writhing, no effect in the first phase of neurogenic pain, and a significant decrease in the second phase of inflammatory pain compared to morphine and aspirin, respectively. Additionally, they found a lack of central (opioid-like) analgesic activity in the salicylate-rich fraction in mice [19]. Alam and colleagues [20] reported the prominent analgesic activity of a salicylate-rich fraction (150 mg/kg) in the acetic acid-induced writhing and tail immersion tests in mice. The tested gaultherin-rich fraction presented relevant inhibition in writhing and an increase in tail-withdrawal latency, compared to diclofenac and tramadol, respectively [20]. A further study conducted by the same research team confirmed the analgesic activity of a salicylate-rich fraction (150 mg/kg) in the tail immersion test in rats, as observed by an increase in the reaction time of tail flicking [21].

### 6.4. Antioxidant Activity

Oxidative stress, characterised by excessive production of reactive oxygen species (ROS), is closely linked to chronic inflammation [122]; therefore, the antioxidant activity of gaultherin has become a research target. The scientists conducted six in vitro non-cellular assays to assess this potential: DPPH and FRAP, two widely used tests based on single electron transfer (SET), and linolenic acid oxidation inhibition, along with hydroxyl, superoxide anion, and hydrogen peroxide scavenging assays, which reflect hydrogen atom transfer (HAT) mechanisms. These evaluations demonstrated that gaultherin exhibits only modest direct antioxidant capacity (Table 5), particularly compared to established synthetic antioxidants, like Trolox^®^, and natural ones, such as quercetin or ascorbic acid [37,38,39,119]. The limited effectiveness of gaultherin in neutralising free radicals is attributed to its low reactivity in both SET and HAT-based reactions [6].

Further research indicated that gaultherin at 0.3–3.0 μg/mL and 25–75 μM significantly and dose-dependently downregulated ROS secretion by LPS-stimulated RAW264.7 murine macrophages in vitro [115] and by *f*MLP-stimulated human neutrophils ex vivo [38,39,119], respectively. Notably, gaultherin exhibited significantly greater antioxidant activity in cell-based models than in non-cellular ROS scavenging assays, where quercetin served as a positive control. This enhanced cellular effect stems from the impact of methyl salicylate glycosides on modulating pro-inflammatory intracellular signalling pathways [117,123].

### 6.5. Molecular Mechanisms of Action

Gaultherin is a prodrug that carries inactive salicylic acid in a glycosylated form, which is enzymatically hydrolysed in the gut to release active salicylate. Once released, salicylic acid exerts anti-inflammatory effects by inhibiting the NF-κB and MAPK pathways and activating the AMPK signalling cascade, thereby modulating key molecular mechanisms involved in inflammation and cellular energy regulation [5,6].

**Figure 3:**
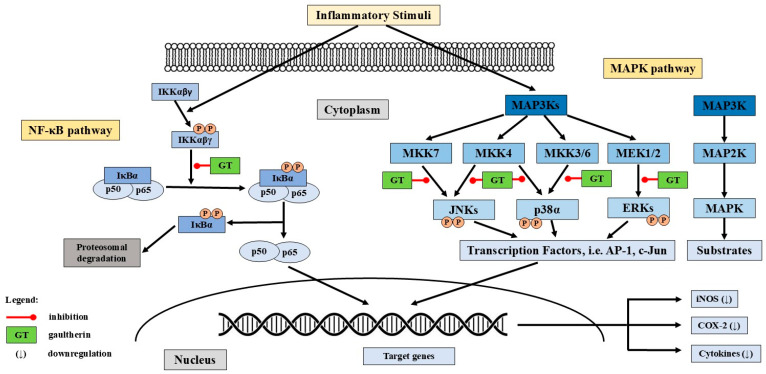
The potential mechanisms of gaultherin’s anti-inflammatory activity. Abbreviations: GT: gaultherin; NF-κB: nuclear factor kappa-light-chain-enhancer of activated B cells; IκBα: nuclear factor of kappa-light-polypeptide gene enhancer in B-cells inhibitor alpha; MAP3K: mitogen-activated protein kinase; MKK/MEK: mitogen-activated protein kinase; JNK: c-Jun *N*-terminal kinases; ERKs: extracellular signal-regulated kinases; AP-1: activator protein-1; c-Jun: transcription factor Jun; iNOS: inducible nitric oxide synthase; COX-2: cyclooxygenase-2; (↓) downregulation.

Gaultherin prevents phosphorylation and subsequent degradation of IκBα, the inhibitory protein that binds to NF-κB and retains it in the cytoplasm (Figure 3). By stabilising IκBα, the translocation of NF-κB to the nucleus is blocked, thereby inhibiting its transcriptional activity. Methyl salicylate glycosides, including gaultherin, reduce the nuclear translocation of the NF-κB p65 subunit, which is critical for initiating the transcription of pro-inflammatory genes. As a result of NF-κB inhibition, the expression of downstream pro-inflammatory cytokines (e.g., TNF-α, IL-1β, IL-6), chemokines, and enzymes (e.g., MMPs, iNOS, COX-2) is significantly reduced [108,115,116,117,118,124].

Salicylates, including aspirin, sodium salicylate, and natural methyl salicylate glycosides, inhibit directly or via upstream modulation of MAPK kinases, particularly MEK1/2, MKK3/6, and MKK4/7, and thus suppress the phosphorylation (activation) of MAPKs, including JNKs (c-Jun *N*-terminal kinases), p38, and ERKs (extracellular signal-regulated kinases) [123]. By damping MAPK signalling, salicylates reduce activation of transcription factors such as AP-1 (Activator Protein-1), c-Jun (transcription factor Jun), and ATF2 (Activating Transcription Factor 2) [125], which blocks expression of pro-inflammatory cytokines like TNF-α, IL-6, and IL-1β [115], and enzymes, e.g., COX-2 and iNOS [126]. While other studies focused on MSL [123,124,127], aspirin [128], and sodium salicylate [129,130], the findings provide insight into the potential mechanisms by which gaultherin might exert anti-inflammatory effects through modulation of the MAPK pathway (Figure 3).

Gaultherin and other methyl salicylate glycosides are structurally related to salicylate compounds such as aspirin, which are known activators of the AMP-activated protein kinase (AMPK) pathway [100,104,131]. Salicylate can directly bind to the AMPK β-subunit and activate the complex independently of AMP and ADP levels. Furthermore, AMPK activation suppresses pathways such as NF-κB and mTOR (mammalian target of rapamycin). This serine/threonine protein kinase regulates cell functions, autophagy, and transcription, thereby contributing to the reduction in inflammation and metabolic dysfunction. While direct evidence for AMPK activation by gaultherin is currently limited, the broader class of salicylate-based compounds, including methyl salicylate derivatives [132], has demonstrated the ability to stimulate AMPK activity in various cell types and tissues.

## 7. Gaultherin vs. Aspirin—Differences in Pharmacokinetic and Pharmacodynamic Effects

The essential differences in pharmacokinetic and pharmacodynamic parameters between gaultherin and aspirin are presented in Table 3.

The main similarities between gaultherin and aspirin concern their pharmacokinetic aspects, such as their prodrug nature and metabolism to salicylic acid, as well as their pharmacodynamic effects, including a common mechanism of biological activity and systemic effects. Gaultherin acts as a natural prodrug, while aspirin is a semi-synthetic prodrug, and both require enzymatic conversion to release the active salicylate. The two are eventually metabolised to salicylic acid, the compound responsible for most of their pharmacological effects. Similarly, both gaultherin and aspirin exert anti-inflammatory, analgesic, and antipyretic effects by inhibiting the COX-2 enzyme, leading to reduced prostaglandin synthesis. After metabolic activation, one and the other release salicylate as the primary active agent, influencing additional signalling pathways such as NF-κB and MAPK, and AMPK cascade (probably also for gaultherin), which mediate inflammation [15,18,100,102,103,104,105,106].

The differences in pharmacokinetic and pharmacodynamic effects between gaultherin and aspirin are, however, more pronounced, which may result in the advantage of the natural methyl salicylate glycoside over the synthetic compound. The first difference appears already at the absorption stage. Aspirin is rapidly absorbed in the stomach and upper small intestine, with peak plasma levels occurring within 30 to 60 min. Gaultherin, a natural salicylate, must first undergo hydrolysis in the gut to release methyl salicylate, which is then absorbed more slowly. In terms of metabolism and bioavailability, aspirin is rapidly hydrolysed by esterases to salicylic acid in the bloodstream and liver. Gaultherin undergoes initial enzymatic cleavage to release methyl salicylate, which is then converted to salicylic acid in the liver, similar to aspirin but with a delayed onset due to the glycosidic bond. Acetylsalicylic acid also has relatively high and predictable oral bioavailability. In contrast, gaultherin’s bioavailability is more variable and generally lower due to its need for enzymatic conversion and slower absorption. Another significant pharmacokinetic difference is the onset of action between the two compounds. Aspirin typically has a faster onset of action compared to gaultherin, which must undergo multiple metabolic steps before becoming pharmacologically active [15,18,100,102,103,104,105,106].

Equally significant are differences in pharmacodynamic effects, i.e., mechanisms of action and duration of therapeutic effect. Both aspirin and gaultherin ultimately act through their active metabolite, salicylic acid, to inhibit COX enzymes. However, aspirin irreversibly inhibits COX-1 and COX-2, while gaultherin inhibits only COX-2 without showing any inhibitory effect on the COX-1 isoform. Due to its non-selective COX inhibition, aspirin has a longer duration of antiplatelet effect. Gaultherin presents a negligible impact on platelet aggregation, as it lacks this irreversible binding [5,6,15,16,18,100,104,108,109].

The methyl salicylate primveroside can also serve as a natural alternative to aspirin, as it presents some relevant advantages over the synthetic compound. Gaultherin’s glycosidic structure prevents direct gastric mucosa irritation, making it less ulcerogenic than aspirin, which directly affects the stomach lining and inhibits protective prostaglandins. Gaultherin is also inactive in the stomach, only becoming activated in the intestine, which reduces systemic exposure peaks and allows for a more gradual release of salicylate, potentially improving tolerability. In addition, unlike aspirin, gaultherin does not irreversibly inhibit platelet function, lowering the risk of bleeding complications in long-term use [5,6,15,16,18,100,104,108,109]. These properties make gaultherin a promising plant-derived prodrug-like compound, capable of delivering methyl salicylate in a safer and more bioavailable form, with targeted modulation of key inflammatory pathways.

## 8. Materials and Methods

The literature search was conducted using well-known databases (Scopus, Web of Science, ScienceDirect, Google Scholar, and PubMed) using the following keyword combination pattern: (1) the chemical or common names of “methyl salicylate xylopyranosyl-glucopyranoside”, or “methyl salicylate xylosyl-glucoside”, or “gaultherin”, or “monotropitoside”, or “monotropitin”, or “methyl salicylate primveroside”; (2) description of the plant part/product (i.e., “whole plant”, or “aerial part/-s”, or “leaf/leaves”, or “stem/-s”, or “fruit/-s”, or “flower/-s”, or “seeds”, or “bark”, or “rhizome/-s”, or “root/-s”, or “extract/-s”, or “infusion/-s”, or “decoction/-s”); (3) names of groups of phenolic constituents (i.e., “salicylates”, or “methyl salicylate/-s”, or “methyl salicylate glycoside/-s”, or “methyl salicylate diglycoside/-s”, or “methyl salicylate triglycoside/-s”, or “methyl salicylate derivatives”); (4) activity descriptor (i.e., “ in vitro”, or “in vivo”, or “ex vivo”, or “in silico”, or “anti-inflammatory”, or “antipyretic”, or “analgesic”, or “antioxidant”, or “activity”, or “toxicity”, or “biocompatibility”, or “inflammation”, or “oxidative stress”); (5) medical or traditional application descriptor (i.e., “medical”, or “traditional”, or “historical”). The databases were searched for original articles written in English, French, German, Chinese, Japanese, Korean, or Russian, and published (at least electronically) up to July 2025. The validation of the items was performed manually (by reading the entire article), and articles with a significant contribution to the field of research are included in the present review.

## 9. Conclusions

Gaultherin is a natural methyl salicylate diglycoside with good bioavailability and a lack of gastric ulcerogenic effect compared to aspirin. The reviewed papers covered over 180 years of phytochemical and biological studies on this salicylate. The researchers have detected gaultherin in over thirty plant species belonging to nineteen botanical families, distributed worldwide, with half of the species from the Ericaceae Juss. and Rosaceae Juss. families. The bioactivity data demonstrated that gaultherin is a safe product with a direct inhibitory potential towards the COX-2 enzyme and an indirect impact on several pathways associated with NF-κB, MAPKs, and potentially AMPK signalling cascade. The beneficial action of this natural methyl salicylate glycoside have been linked to lack of direct inhibition of the constitutive COX-1 isoform, which results in the cytoprotective effects of E2 and I2 prostaglandins on the gastric mucosa (no ulcerogenic impact), and no inhibition of platelet aggregation which could consequently cause significant side effects, such as bleeding ulcers or intracranial haemorrhage, observed when using aspirin. The conducted animal studies indicated a strong and positive impact of gaultherin on croton oil-induced ear oedema, carrageenan-induced paw oedema, hot plate-induced pain, Brewer’s yeast-induced pyrexia, acetic acid-induced writhing, and acetic acid-induced abdominal contractions, and thus presented significant anti-inflammatory, antipyretic, and analgesic potential. Scientists have suggested gaultherin as a promising agent for preventing or treating inflammation-related disorders, including rheumatoid arthritis, swelling, sciatica, neuralgia, muscular pain, and other types of pain of various aetiologies. Although cell-based tests and animal models have demonstrated the biocompatibility of gaultherin to non-tumorigenic cells and tissues, further research should focus on additional cytotoxicity studies. So far, only one proposed isolation technique applied a simple one-step extraction of the plant material and a high-performance preparative chromatographic method, allowing the obtaining of gaultherin in its pure crystalline form (not as a fraction rich in salicylates) with satisfactory isolation yield. However, the widespread study of gaultherin and its clinical trials is hindered primarily by its high market cost and the absence of laboratory-scale applications for the developed isolation method. Therefore, the development of new isolation techniques that will allow for the obtaining of gaultherin in its pure form should become the goal of extended phytochemical studies. The entire *Gaultheria procumbens* plant (including its fruits, leaves, and stems), as well as the leaves of other *Gaultheria* species, appear to be the most viable options for the large-scale and cost-efficient extraction of gaultherin. Increasing the availability of gaultherin in this way would facilitate further in vivo research and practical application studies. Moreover, while the therapeutic potential of gaultherin is promising, significant gaps remain in its path toward clinical translation. Currently, there is a lack of robust clinical trials evaluating its efficacy and safety in humans. The transition from preclinical data to human applications requires addressing regulatory approval processes, which may be complicated by the compound’s classification as a natural product and the limited precedent for its pharmaceutical use. Additionally, the high production cost—whether through extraction or synthesis—poses a challenge for commercial viability and patient access. Addressing these barriers through coordinated research efforts, regulatory planning, and cost-reduction strategies will be essential for advancing gaultherin from experimental findings to a clinically applicable therapeutic agent.

## Figures and Tables

**Table 4:** Cellular safety of gaultherin and other methyl or ethyl salicylate glycosides.

Compound	Cell Model	Concentration Tested	Safe Concentration	Method	References
gaultherin	human neutrophils	25, 50, 75 μM	25–75 μM	flow cytometry with propidium iodide staining, 24 h	[37,38]
	RAW264.7 murine macrophages	–	0.1–100 μg/mL	MTT, 24 h	[115]
MSL	primary rat glial cells (microglia and astrocytes)	0.1, 1, 10 μM	0.1–10 μM	MTT, 24 h	[116]
	RAW264.7 murine macrophages	–	0.01–100 μM	MTT, 48 h	[117]
	RAW264.7 murine macrophages	1, 3, 10, 30, 100, 300, 600, 800, 1000 μM	1–1000 μM	MTT, 24 h	[118]
ethyl salicylate 2-*O*-β-D-glucoside	RAW264.7 murine macrophages	2, 10, 50, 100 μM	2–100 μM	MTT, 48 h	[108]
PH	human neutrophils	25, 50, 75 μM	25–75 μM	flow cytometry with propidium iodide staining, 24 h	[37]
MSTG-A	RAW264.7 murine macrophages	–	0.1–100 μg/mL	MTT, 24 h	[115]
MSTG-B	human neutrophils	25, 50, 75 μM	25–75 μM	flow cytometry with propidium iodide staining, 24 h	[37]

MSL: methyl salicylate 2-*O*-β-D-lactoside; PH: methyl salicylate 2-*O*-β-D-glucopyranosyl-(1→2)-β-D-glucopyranoside (physanguloside A); MSTG-A: methyl salicylate 2-*O*-β-D-xylopyranosyl-(1→2)-[β-D-xylopyranosyl-(1→6)]-β-D-glucopyranoside; MSTG-B: methyl salicylate 2-*O*-β-D-glucopyranosyl-(1→2)-[β-D-xylopyranosyl-(1→6)]-β-D-glucopyranoside; MTT assay: 3-(4,5-dimethylthiazol-2-yl)-2,5-diphenyltetrazolium bromide assay.

**Table 5:** Biological activity of gaultherin and gaultherin-rich plant fractions.

Disease/Pathology Model	Type of Study	Type of Groups, Treatment	Effect of Gaultherin Treatment	References
Antioxidant activity				
Oxidative stress-related disorders	In vitro studies	Positive controls: DPPH; QU: SC_50_ = 1.65 μg/mL; TX: SC_50_ = 4.31 μg/mL; FRAP; QU: 47.09 mmol Fe^2+^/g; TX: 11.89 mmol Fe^2+^/g; TBARS; QU: IC_50_ = 1.78 μg/mL; TX: IC_50_ = 4.68 μg/mL; O_2_^•−^; QU: SC_50_ = 7.58 μg/mL; TX: SC_50_ = 135.24 μg/mL; ^•^OH; QU: SC_50_ = 42.48 μg/mL; TX: SC_50_ = 165.45 μg/mL; H_2_O_2_; QU: SC_50_ = 7.52 μg/mL; TX: SC_50_ = 15.87 μg/mL	Gaultherin presented low antioxidant activity; DPPH: SC_50_ = 265.69 μg/mL; FRAP: 0.64 mmol Fe^2+^/g; TBARS: IC_50_ = 269.40 μg/mL; O_2_^•−^: SC_50_ = 451.76 μg/mL; ^•^OH: SC_50_ = 488.52 μg/mL; H_2_O_2_: SC_50_ = 587.86 μg/mL	[37,39,119]
		Positive controls: FRAP; AA: 3.97 mol/mol, TX: 2.98 mol/mol; O_2_^•−^; AA: SC_50_ = 29.87 μM, TX: SC_50_ = 540.33 μM	Gaultherin presented low ferric reducing activity and scavenging potential towards O_2_^•−^; FRAP: 0.29 mol/mol; O_2_^•−^: 1012.00 μM	[38]
	In vitro study of LPS-stimulated RAW264.7 murine macrophages; Impact on ROS level	–	Gaultherin exhibited weak activity towards ROS secretion; ROS level: 88.75% (gaultherin concentration 0.3 μg/mL), 56.25% (1.0 μg/mL), 47.5% (3.0 μg/mL)	[115]
	Ex vivo studies in *f*MLP-stimulated human neutrophils; Impact on ROS level	Positive control: ROS level; QU: 48.2% (25 μM), 25.6% (50 μM), 20.4% (75 μM)	Gaultherin exhibited weak activity towards ROS secretion; ROS level: 78.5% (gaultherin concentration 25 μM), 55.2% (50 μM), 47.7% (75 μM)	[38,39,119]
Anti-inflammatory activity				
Inflammation-related disorders	In vitro studies; Inhibition of hyaluronidase (HYAL), lipoxygenase (LOX), and cyclooxygenase-2 (COX-2) activity	Positive controls: HYAL; IND: IC_50_ = 12.77 μg/mL; DEX: IC_50_ = 14.18 μg/mL; QU: IC_50_ = 30.78 μg/mL; LOX; IND: IC_50_ = 0.09 mg/mL; DEX: IC_50_ = 0.12 mg/mL; QU: IC_50_ = 0.09 mg/mL; COX-2; IND: IC_50_ = 0.18 mg/mL; DEX: IC_50_ = 0.51 mg/mL; QU: IC_50_ = 0.47 mg/mL	Gaultherin presented moderate anti-inflammatory activity towards hyaluronidase and lipoxygenase and significant activity against cyclooxygenase-2 enzymes; HYAL; IC_50_ = 28.58 μg/mL; LOX; IC_50_ = 0.56 mg/mL; COX-2; IC_50_ = 0.35 mg/mL	[37,39,119]
	In vitro studies; Inhibition of hyaluronidase (HYAL) and cyclooxygenase-2 (COX-2) activity	Positive controls: HYAL; IND: IC_50_ = 35.69 μM; DEX: IC_50_ = 36.13 μM; COX-2; IND: IC_50_ = 0.50 mM; DEX: IC_50_ = 1.29 mM	Gaultherin presented moderate anti-inflammatory activity towards hyaluronidase and significant activity against cyclooxygenase-2 enzymes; HYAL; IC_50_ = 64.02 μM; COX-2; IC_50_ = 0.78 mM	[38]
	Ex-vivo study of LPS/*f*MLP+cytochalasin B-stimulated human neutrophils	Positive controls: IL-8; DEX: 55.8% (25 μM), 67.8% (50 μM), 76.9% (75 μM); IL-1β; DEX: 51.8% (25 μM), 74.4% (50 μM), 79.6% (75 μM); TNF-α; DEX: 68.8% (25 μM), 91.4% (50 μM), 98.7% (75 μM); MMP-9; DEX: 26.9% (25 μM), 30.2% (50 μM), 43.7% (75 μM); ELA-2; QU: 39.3% (25 μM), 47.0% (50 μM), 55.6% (75 μM)	Gaultherin significantly ↓ the release of pro-inflammatory cytokines; IL-8; % of inhibition 0% (gaultherin concentration 25 μM), 14.2% (50 μM), 25.6% (75 μM); IL-1β; 13.7% (25 μM), 32.4% (50 μM), 46.6% (75 μM); TNF-α; 6.0% (25 μM), 48.9% (50 μM), 73.7% (75 μM); MMP-9; 6.3% (25 μM), 22.9 (50 μM), 35.6% (75 μM); ELA-2; 34.1% (25 μM), 54.8% (50 μM), 64.9% (75 μM)	[37,38,39,119]
	In vitro studies; Inhibition of hyaluronidase (HYAL)	Positive controls: HYAL; RA: 41.3% (positive control concentration 1.0 mM); IC_50_ = 1.36 mM	Gaultherin presented weak anti-inflammatory activity towards the hyaluronidase enzyme; HYAL; % of inhibition 0.6% (gaultherin concentration 1.0 mM)	[48]
	In vitro studies; Inhibition of lipoxygenase (5-LOX) and COX-2	Positive controls: 5-LOX; ZIL: IC_50_ = 12.00 μg/mL; COX-2; CX: IC_50_ = 10.00 μg/mL	Salicylate-rich fraction (containing gaultherin) exhibited a weak direct ability to inhibit pro-inflammatory enzymes. 5-LOX; IC_50_ = 39.70 μg/mL; COX-2; IC_50_ = 77.20 μg/mL	[20]
	In vitro study of LPS-stimulated RAW264.7 murine macrophages; Inhibition of NO production	Positive control: NO; HYD: IC_50_ = 58.79 μM	Gaultherin presented weak anti-inflammatory activity toward nitric oxide production; NO; IC_50_ > 100 μM	[83]
	In vitro study of LPS-stimulated RAW264.7 murine macrophages; Inhibition of NO production	Positive control: NO; freshly cultured medium: Inhibition [%]: -	Gaultherin moderately ↓ the production of nitric oxide; NO; Inhibition [%]: 39.8	[34]
	In vitro study of LPS-stimulated RAW264.7 murine macrophages; Inhibition of pro-inflammatory cytokines release and NO production	–	Gaultherin significantly ↓ the release of pro-inflammatory cytokines and production of NO; TNF-α; % of inhibition 11.21% (gaultherin concentration 0.3 μg/mL), 32.69% (1.0 μg/mL), 56.46% (3.0 μg/mL); IL-1β; 53.58% (0.3 μg/mL), 64.40% (1.0 μg/mL), 75.67% (3.0 μg/mL); IL-6; 61.81% (0.3 μg/mL), 71.26% (1.0 μg/mL), 73.15% (3.0 μg/mL); NO; 22.97% (0.3 μg/mL), 45.90% (1.0 μg/mL), 56.20% (3.0 μg/mL)	[115]
Carrageenan-induced paw oedema	In vivo animal model; Albino mice of both genders; n = 6/group	1. Normal control (saline); 2. Diclofenac group (10 mg/kg); 3–5. Groups treated with the plant extract in a dose of 100, 200, and 300 mg/kg, respectively; 6. Group treated with a salicylate-rich fraction (150 mg/kg) containing gaultherin; Inhibition [%]; diclofenac: 19.17 (1 h), 32.87 (2 h), 56.6 (3 h), 61.6 (4 h), 76.7 (5 h)	Salicylate-rich fraction ↓ the carrageen-induced paw oedema with a maximum inhibition of 67.75%, after the standard drug diclofenac with 76.7% inhibition; Inhibition [%]; salicylate rich fraction: 15.06 (1 h), 15.06 (2 h), 34.24 (3 h), 52.05 (4 h), 65.75 (5 h)	[20]
Croton oil-induced ear oedema		1. Normal control (saline); 2. Celecoxib group (100 mg/kg); 3–5. Groups treated with the plant extract in a dose of 100, 200, and 300 mg/kg, respectively; 6. Group treated with salicylate-rich fraction (150 mg/kg) containing gaultherin; Oedema [mg]; celecoxib: 3.9; Inhibition [%]; celecoxib: 70.2	Salicylate-rich fraction ↓ the croton oil-induced ear oedema and showed almost similar and significant (*p* < 0.01) anti-inflammatory effect as the standard drug celecoxib; Oedema [mg]; salicylate-rich fraction: 4.5; Inhibition [%]; salicylate-rich fraction: 65.7	[20]
Croton oil-induced ear oedema	In vivo animal model; Kunming mice—males; n = 10/group	1. Normal control; 2. Aspirin group (200 mg/kg); 3. Gaultherin group (400 mg/kg) Oedema [g]; aspirin: 10.8; Inhibition [%]: 44	Gaultherin significantly ↓ the ear swelling after application of croton oil; Oedema [g]; gaultherin: 11.8; Inhibition [%]: 39	[15]
Croton oil-induced ear oedema	In vivo animal model; Kunming mice of both genders; n = 10/group	1. Normal control; 2. Aspirin group (200 mg/kg); 3–5. Groups treated with the salicylate-rich fraction in a dose of 200, 400, and 800 mg/kg, respectively, containing 50% of gaultherin; Oedema [mg]; aspirin: 6.39; Inhibition [%]: 48.75	Salicylate-rich fraction significantly ↓ of ear oedema with topical application of croton oil; Oedema [mg]; salicylate-rich fraction: 8.11 (200 mg/kg), 7.07 (mg/kg), 6.44 (800 mg/kg); Inhibition [%]: 34.94 (200 mg/kg), 43.29 (400 mg/kg), 48.3 (800 mg/kg)	[19]
Carrageenan-induced paw oedema	In vivo animal model; Wistar rats of both genders; n = 8/group	1. Normal control; 2. Indomethacin group (10 mg/kg); 3–5. Groups treated with the salicylate derivatives fraction in a dose of 100, 200, and 400 mg/kg, respectively, containing 50% of gaultherin; Paw oedema [mm]; indomethacin: 2.94 (1 h), 2.31 (2 h), 2.75 (4 h), 3.19 (6 h)	Salicylate derivatives fraction significantly ↓ the carrageenan-induced oedema in 6 h measurements; Paw oedema [mm]; salicylate derivatives fraction: 100 mg/kg: 3.94 (1 h), 3.63 (2 h), 4.94 (4 h), 5.19 (6 h); 200 mg/kg: 2.94 (1 h), 3.44 (2 h), 3.94 (4 h), 3.31 (6 h); 400 mg/kg: 2.75 (1 h), 2.63 (2 h), 3.31 (4 h), 3.06 (6 h)	[19]
Ankle joint diameter measurements	In vivo animal model; Albino rats of both genders; n = 6/group	1. Normal control; 2. Negative control; 3. Diclofenac group (10 mg/kg); 4–6. Groups treated with the plant extract in a dose of 100, 200, and 300 mg/kg, respectively; 7. Group treated with a salicylate-rich fraction (150 mg/kg) containing gaultherin;Tail response time [s]; diclofenac: 11.50 (30 min), 13.20 (60 min), 14.5 (120 min)	Salicylate-rich fraction showed a moderate ↓ in joint diameter after 12 days of the experiment	[21]
Biochemical parameters in blood	In vivo animal model; Albino rats of both genders; n = 6/group	1. Normal control; 2. Negative control; 3. Diclofenac group (10 mg/kg); 4–6. Groups treated with the plant extract in a dose of 100, 200, and 300 mg/kg, respectively; 7. Group treated with a salicylate-rich fraction (150 mg/kg) containing gaultherin; Diclofenac; AST: 92 (U/L); ALT: 106 (U/L); ALP: 299 (U/L); Total protein: 5.94 (g/dL)	Salicylate-rich fraction significantly ↓ the concentration of AST, ALT, and ALP. In addition, there was a significant ↑ in the level of total protein in the blood; AST: 105 (U/L); ALT: 123 (U/L); ALP: 349 (U/L); Total protein: 5.73 (g/dL)	[21]
Antipyretic activity				
Brewer’s yeast-induced pyrexia	In vivo animal model; Albino mice of both genders; n = 6/group	1. Normal control (saline); 2. Paracetamol group (150 mg/kg); 3–5. Groups treated with the plant extract in a dose of 100, 200, and 300 mg/kg, respectively; 6. Group treated with salicylate-rich fraction (150 mg/kg) containing gaultherin; Rectal temperature [°C]; paracetamol: 38.2 (0 h), 39.1 (1 h), 39.2 (2 h), 39.1 (3 h), 38.1 (4 h), 37.0 (5 h)	Salicylate rich fraction ↓ the pyrexia significantly in the 5th hour of experiment (*p* < 0.01); Rectal temperature [°C]; salicylate-rich fraction: 38.6 (0 h), 38.8 (1 h), 38.9 (2 h), 38.2 (3 h), 37.5 (4 h), 37.2 (5 h)	[20]
Analgesic activity				
Acetic acid-induced abdominal contraction	In vivo animal model; Kunming mice—males; n = 10/group	1. Normal control; 2. Aspirin group (200 mg/kg); 3. Gaultherin group (400 mg/kg) Number of contractions; aspirin: 17.6; Inhibition [%]: 44	Gaultherin significantly ↓ the visceral pain induced by acetic acid; Number of contractions; gaultherin: 21.0; Inhibition [%]: 33	[15]
Ulcerogenic effect on the gastric mucosa	In vivo animal model; Wistar rats—males; n = 6–8/group	1. Normal control; 2. Aspirin group (135 mg/kg); 3. Gaultherin group (400 mg/kg) Gastric lesion [mm^2^]: aspirin: 20.3	Gaultherin caused no lesions in the rat stomachs; Gastric lesion [mm^2^]: gaultherin: 0	[15]
Acute gastric lesions induced by water immersion restrain stress	In vivo animal model; Wistar rats—males; n = 6–8/group	1. Normal control; 2. Aspirin group (135 mg/kg); 3. Gaultherin group (330 mg/kg) Gastric lesion [mm^2^]: aspirin: 50.6	Gaultherin did not significantly affect the ulcerogenic response to water immersion restraint stress; Gastric lesion [mm^2^]: gaultherin: 31.1	[15]
Hot plate-induced pain	In vivo animal model; Kunming mice of both genders; n = 10/group	1. Normal control; 2. Morphine group (5 mg/kg); 3–4. Groups treated with the salicylate-rich fraction in a dose of 400 and 800 mg/kg, respectively, containing 50% of gaultherin; Latency [s]; morphine: 0 h: 19.2; 2 h: 33.9; 3 h: 47.3; 4 h: 40.0	Salicylate-rich fraction did not alter the latency reaction to the thermal stimulus, as demonstrated by the hot plate test; Latency [s]; salicylate-rich fraction: 0 h: 17.9 (400 mg/kg), 17.3 (800 mg/kg); 2 h: 18.1 (400 mg/kg), 22.5 (800 mg/kg); 3 h: 18.6 (400 mg/kg), 23.4 (800 mg/kg); 4 h: 22.4 (400 mg/kg), 23.0 (800 mg/kg)	[19]
Acetic acid-induced writhing	In vivo animal model; Kunming mice of both genders; n = 10/group	1. Normal control; 2. Aspirin group (200 mg/kg); 3–5. Groups treated with the salicylate-rich fraction in a dose of 200, 400, and 800 mg/kg, respectively, containing 50% of gaultherin; Number of writhing; aspirin: 6.67	Salicylate-rich fraction significantly ↓ the number of writhing and stretching induced by the acetic acid; Number of writhing; salicylate-rich fraction: 15.75 (200 mg/kg), 14.73 (400 mg/kg), 8.83 (800 mg/kg)	[19]
Formalin-induced pain	In vivo animal model; Kunming mice of both genders; n = 10/group	1. Normal control; 2. Aspirin group (200 mg/kg); 3. Morphine group (5 mg/kg); 4–6. Groups treated with the salicylate-rich fraction in a dose of 200, 400, and 800 mg/kg, respectively, containing 50% of gaultherin; Paw licking time [s]; aspirin: 0–5 min: 56.1; Inhibition [%]: −2.8; 20–30 min: 95.9; Inhibition [%]: 40.2; morphine: 0–5 min: 16.7; Inhibition [%]: 69.4; 20–30 min: 88.3; Inhibition [%]: 44.9	Salicylate-rich fraction exerted no effect on the first phase (0–5 min) of the formalin test; however, in the second phase (20–30 min), it significantly ↓ the time of paw licking; Paw licking time [s]; salicylate-rich fraction: 0–5 min: 55.1 (200 mg/kg), 54.7 (400 mg/kg), 53.5 (800 mg/kg); Inhibition [%]: −0.9 (200 mg/kg), −0.1 (400 mg/kg), 2.0 (800 mg/kg); 20–30 min: 144.4 (200 mg/kg), 95.5 (400 mg/kg), 93.3 (800 mg/kg); Inhibition [%]: 9.9 (200 mg/kg), 40.4 (400 mg/kg), 41.8 (800 mg/kg)	[19]
Acetic acid-induced writhing test	In vivo animal model; Albino mice of both genders; n = 6/group	1. Normal control (saline); 2. Diclofenac group (10 mg/kg); 3–5. Groups treated with the plant extract in a dose of 100, 200, and 300 mg/kg, respectively; 6. Group treated with a salicylate-rich fraction (150 mg/kg) containing gaultherin; Number of writhing (20 min); diclofenac: 18.46	Salicylate-rich fraction ↓ the analgesia to a significant level (*p* < 0.01), and the result was comparable to diclofenac used as a standard drug; Number of writhing (20 min); salicylate-rich fraction: 27.66	[20]
Tail immersion test		1. Normal control (saline); 2. Tramadol group (30 mg/kg); 3–5. Groups treated with the plant extract in a dose of 100, 200, and 300 mg/kg, respectively; 6. Group treated with a salicylate-rich fraction (150 mg/kg) containing gaultherin; Tail response time [s]; tramadol: 3.28 (0 min), 3.27 (30 min), 4.09 (60 min), 5.50 (90 min), 6.63 (120 min)	Salicylate-rich fraction ↑ the tail immersion time (↑ the tail-withdrawal latency) to a significant level (*p* > 0.01) after 90 min, and the result was comparable to tramadol used as a standard drug; Tail response time [s]; salicylate-rich fraction: 3.21 (0 min), 3.50 (30 min), 3.71 (60 min), 4.87 (90 min), 4.95 (120 min)	[20]
Tail immersion test	In vivo animal model; Albino rats of both genders; n = 6/group	1. Normal control; 2. Negative control; 3. Diclofenac group (10 mg/kg); 4–6. Groups treated with the plant extract in a dose of 100, 200, and 300 mg/kg, respectively; 7. Group treated with a salicylate-rich fraction (150 mg/kg) containing gaultherin; Tail response time [s]; diclofenac: 11.50 (30 min), 13.20 (60 min), 14.5 (120 min)	Salicylate-rich fraction significantly ↑ the tail immersion time after 60 min, and the result was comparable to diclofenac used as a standard drug; Tail response time [s]; salicylate-rich fraction: 9.77 (30 min), 11.83 (60 min), 13.26 (120 min)	[21]
Pain haematological parameters in blood	In vivo animal model; Albino rats of both genders; n = 6/group	1. Normal control; 2. Negative control; 3. Diclofenac group (10 mg/kg); 4–6. Groups treated with the plant extract in a dose of 100, 200, and 300 mg/kg, respectively; 7. Group treated with a salicylate-rich fraction (150 mg/kg) containing gaultherin; Diclofenac; RBC: 4.93 (10^6^ cells/mm^3^); WBC: 10.01 (10^3^ cells/mm^3^); Hb: 13.1 (g/dL); Platelets: 1328 (10^3^ cells/mm^3^); CRP: 3.90 (mg/lit); RF value: 43 (IU/mL)	Salicylate-rich fraction significantly ↓ the C-reactive protein and rheumatoid factor in the blood; RBC: 4.60 (10^6^ cells/mm^3^); WBC: 11 (10^3^ cells/mm^3^); Hb: 12.4 (g/dL); Platelets: 1468 (10^3^ cells/mm^3^); CRP: 4.73 (mg/lit); RF value: 49 (IU/mL)	[21]

AA: ascorbic acid; TX: Trolox^®^ (6-hydroxy-2,5,7,8-tetramethylchroman-2-carboxylic acid); DEX: dexamethasone; HYD: hydrocortisone; QU: quercetin; ZIL: zileuton; CX: celecoxib; RA: rosmarinic acid; DPPH: DPPH^•^ radical scavenging test; FRAP: antioxidant activity expressed in millimoles of Fe^2+^ ions/g of tested compound (Ferric Reducing Antioxidant Power); TBARS: linoleic acid oxidation inhibition test that measures the level of thiobarbituric acid reactive substances produced; O_2_^•−^: superoxide radical anion scavenging test; ^•^OH: hydroxyl radical scavenging test; H_2_O_2_: hydrogen peroxide reduction test; ROS: Reactive Oxygen Species; HYAL: hyaluronidase inhibition assay; LOX: lipoxygenase inhibition assay; COX-2: cyclooxygenase-2 inhibition assay; IL: interleukin; TNF-α: tumour necrosis factor-α (tumour necrosis factor-α); MMP-9: matrix metalloproteinase-9; ELA-2: human elastase-2; NO: nitric oxide inhibition assay; LPS: bacterial lipopolysaccharide obtained from *Escherichia coli* O111:B4; *f*MLP: *N*-formyl-L-methionyl-L-leucyl-L-phenylalanine; IC_50_: concentration of a tested extract or reference substance, expressed in μg/mL or mg/mL, which reduces the enzyme activity or inhibits the oxidation of linolenic acid by 50% (*Inhibitory Concentration*); (↑) upregulation; (↓) downregulation.

## Abbreviations

AA	ascorbic acid
AMPK	AMP-activated protein kinase
Akt	protein kinase B (PKB)
AP-1	activator protein-1
ATF2	activating transcription factor 2
c-Jun	transcription factor Jun
COX	cyclooxygenase
CX	celecoxib
DEX	dexamethasone
dw	dry weight
ELA-2	human elastase-2
ERK	extracellular signal-regulated kinases
*f*MLP	*N*-formyl-L-methionyl-L-leucyl-L-phenylalanine
H_2_O_2_	hydrogen peroxide
HYAL	hyaluronidase
HYD	hydrocortisone
IL	interleukin
IκBα	nuclear factor of kappa-light-polypeptide gene enhancer in B-cells inhibitor alpha
iNOS	inducible nitric oxide synthase
JNK	c-Jun *N*-terminal kinases
LPS	bacterial lipopolysaccharide from *Escherichia coli* O55:B5
LOX	lipoxygenase
MAPK	mitogen-activated protein kinase
MMP-9	matrix metalloproteinase-9
MSL	methyl salicylate 2-*O*-β-D-lactoside
MSTG-A	methyl salicylate 2-*O*-β-D-xylopyranosyl-(1→2)-[β-D-xylopyranosyl-(1→6)]-β-D-glucopyranoside
MSTG-B	methyl salicylate 2-*O*-β-D-glucopyranosyl-(1→2)-[β-D-xylopyranosyl-(1→6)]-β-D-glucopyranoside
mTOR	mammalian target of rapamycin
MTT	3-(4,5-dimethylthiazol-2-yl)-2,5-diphenyltetrazolium bromide
NF-κB	nuclear factor kappa-light-chain-enhancer of activated B cells
NSAIDs	nonsteroidal anti-inflammatory drugs
NO	nitric oxide
O_2_^•−^	superoxide anion radical
^•^OH	hydroxyl radical
QU	quercetin
P-gp	P-glycoprotein
PH	methyl salicylate 2-*O*-β-D-glucopyranosyl-(1→2)-β-D-glucopyranoside (physanguloside A)
PI3K	phosphoinositide 3-kinase
RA	rosmarinic acid
ROS	reactive oxygen species
TNF-α	tumour necrosis factor-α
TX	Trolox^®^ (6-hydroxy-2,5,7,8-tetramethylchroman-2-carboxylic acid)
ZIL	zileuton
